# Red Box, Green Box: Psychometric evaluation of a self‐report behavioral frequency measurement approach for behavioral addictions research

**DOI:** 10.1111/add.70192

**Published:** 2025-09-26

**Authors:** Matthew W. R. Stevens, Marcela Radünz, Christina Galanis, Blake Quinney, Ian Zajac, Joël Billieux, Paul H. Delfabbro, Daniel L. King

**Affiliations:** ^1^ College of Education, Psychology and Social Work Flinders University Adelaide Australia; ^2^ School of Biomedicine University of Adelaide Adelaide Australia; ^3^ School of Psychology University of Adelaide Adelaide Australia; ^4^ Institute of Psychology University of Lausanne Lausanne Switzerland; ^5^ Centre for Excessive Gambling, Addiction Medicine Lausanne University Hospital (CHUV) Lausanne Switzerland

**Keywords:** addiction, behavior, gaming disorder, ICD‐11, measurement, tool

## Abstract

**Aims:**

The behavioral addictions field lacks clinically useful behavior frequency measures. This study evaluated the psychometric performance of the new ‘Red Box, Green Box’ method for measuring gaming behavior with a focus on its utility for gaming disorder (GD) screening.

**Design, setting and participants:**

A prospective, cross‐sectional survey study was conducted using an online crowdsourcing platform. Participants were 1149 male gamers aged 18–35 years from Australia, Canada, United States, United Kingdom and Asia, reporting ≥12 hours of weekly gaming.

**Measurements:**

Gaming time was measured using a conventional weekly hours item, Red Box hours (gaming instead of fulfilling responsibilities) and Green Box hours (gaming during free or leisure time). GD was assessed by the Internet Gaming Disorder Test (IGDT‐10), with *International Classification of Diseases*, 11th Revision (ICD‐11) and *Diagnostic and Statistical Manual of Mental Disorders*, Fifth Edition, Text Revision (DSM‐5‐TR) scoring approaches. Psychological distress [Depression, Anxiety and Stress Scale ‐ 21 Items (DASS‐21)] and impulsivity [Barratt Impulsiveness Scale–short form (BIS‐15)] were measured.

**Findings:**

Gamers with GD reported higher Red Box hours [mean (*M*) = 21.1, standard deviation (SD) = 11.3] than those without (*M* = 8.7, SD = 8.4; *P* < 0.001), and greater Red Box proportion (41.9% vs. 26.8%; *P* < 0.001). Red Box hours demonstrated superior diagnostic accuracy for GD [area under the curve (AUC) = 0.86, sensitivity = 0.94, specificity = 0.63] and Internet gaming disorder (IGD) (AUC = 0.76, sensitivity = 0.88, specificity = 0.56), outperforming comparative measures. A Red Box response of ≥ 9.5 hours had a 94% likelihood of indicating ICD‐11 GD.

**Conclusions:**

The ‘Red Box, Green Box’ method appears to effectively identify *International Classification of Diseases*, 11th Revision, gaming disorder risk among males. Red Box hours demonstrated greater classification validity than the conventional weekly hours approach. This method provides a simple tool for epidemiological research, routine screening (e.g. outpatient consultation) and clinical assessment and treatment planning. Further validation in clinical populations and longitudinal studies is needed.

## INTRODUCTION

Video gaming is a globally popular activity that many find time for in between other important responsibilities and daily living tasks. While most individuals play games in psychologically adaptive ways and/or without associated risks, a minority of players play games persistently with resultant negative psychological, physical, social and other effects [1]. Decades of research on the phenomenon of problem gaming led to the World Health Organization's official inclusion of gaming disorder (GD) and hazardous gaming (HG) categories in the International Classification of Diseases 11th Revision (ICD‐11) [2]. Prevalence estimates of GD based on self‐report symptom checklist approaches suggest that 1% to 2% of gamers worldwide meet the criteria [3, 4, 5]. Despite this recognition, a challenge for the GD field is how to measure behavior patterns that may indicate high probability of health risk and functional impairment [6, 7]. Conventional measurement approaches have involved assessing total time investment in gaming (e.g. hours per week), but this basic indicator lacks contextual information and tends to be only moderately related to problem gaming [8, 9]. Accordingly, the aim of this psychometric investigation was to critically evaluate a new approach to measuring gaming behavior, termed the ‘Red Box, Green Box’ method [10], including its potential utility for screening GD risk.

The growing clinical significance of problematic gaming is evidenced by its formal recognition in two major health classification systems. The most recent Diagnostic and Statistical Manual of Mental Disorders (DSM‐5‐TR) includes internet gaming disorder (IGD) as a condition for further study. As an addictive disorder, the condition is characterized by features including loss of control, tolerance and withdrawal and functional impairment because of gaming. Similarly, the ICD‐11 recognizes GD as a condition characterized by loss of control, prioritization of gaming over important activities and continued use despite harm [11]. Unlike other addiction fields in which research practices have coalesced around a select few tools for screening purposes (e.g. the ASSIST in substance use research), the GD field has become complicated by its use of more than 30 different GD tools [8]. Many GD screeners have burdensome length (i.e. most are 9–20 items) and refer to concepts (e.g. craving, preoccupation and tolerance) that can be unusual and off‐putting for some respondents. Especially in clinical practice, some clients may not endorse some or all items on self‐report GD symptom checklists [12, 13] for reasons including misunderstanding, biases or misgivings about gaming addiction (e.g. adolescents who disagree or deny that gaming can be harmful). Therefore, there is a need for simple complementary tools that: (1) can effectively identify risk and harm; (2) may be less susceptible to misinterpretation or bias (e.g. anti‐GD bias, lack of insight); and (3) have relevance and utility for public health objectives (e.g. education using a simple indicator of unhealthy use).

The most common measure of gaming‐related risk and harm adjacent to GD screening tools has been an individual's overall gaming involvement (i.e. ‘screen time’). Since the 1990s, this conventional approach has involved asking respondents to report their typical involvement in gaming, usually hours per week or, less commonly, hours per gaming session. This method is used in population surveys, and treatment studies often use gaming time as an inclusion criterion and measure of treatment outcome [14, 15, 16]. Aside from the issue that this method tends to yield inaccurate data [17], its main drawback is that some individuals can be quite highly involved in gaming (e.g. gaming for 30 hours per week) without experiencing any problems (i.e. gaming time is not inherently problematic) [18, 19, 20]. High engagement in gaming is common among certain demographic groups, as well as for those who work in the gaming industry, including professional gamers (e.g. eSports players and streamers) [21, 22]. Another issue is total gaming time data fails to capture the context and is not calibrated to the individual's circumstances. Some research has proposed more accurate, but labor‐intensive data collection methods, for example, ecological momentary assessment [23] and telemetry data from online game servers [24], but the field would benefit from simple and practical behavioral measures attuned to the context and functional consequences of gaming [25, 26].

The Red Box, Green Box method [10] was proposed as a refinement of conventional self‐report behavior questions. The method delineates gaming behaviors into two main types: (1) time spent gaming during ‘free’ or leisure time (Green Box); and (2) time spent gaming at times when the respondent should be doing something else, for example, studying, working, sleeping, exercising (Red Box). With this approach, the method aims to calibrate behavioral frequency to each respondent and yield insights into displacement effects associated with frequency of use. Another advantage of the measure is that it is quick to administer and avoids reference to addiction concepts or gaming harms that, for some respondents, may be misunderstood [27], interpreted as an anti‐gaming value judgement or otherwise elicit denial [28]. The Red Box, Green Box measure is beginning to be used in population studies currently underway, but the method has not yet been psychometrically examined. Although the measure is not intended as a standalone measure of problem gaming—but, instead to complement other screening measures and have utility in non‐clinical populations—it is worthwhile to investigate its diagnostic accuracy, sensitivity and specificity compared to routinely used GD measures.

Psychometric evaluation in addiction research should consider some of the common individual differences that affect test performance. Like other addictive disorders, clinically significant gaming problems are influenced by individual differences in impulsivity and psychological distress. Impulsivity, an established risk factor in addictive behaviors [29, 30], predisposes individuals to unregulated gaming by impairing decision‐making and prioritizing immediate rewards over long‐term goals [31]. Studies have linked heightened motor, non‐planning and attentional impulsivity to increased gaming severity, suggesting a predisposition to compulsive play [32, 33]. Additionally, individuals with higher psychological distress are more likely to exhibit addictive behaviors [34]. Mood disorders can precipitate excessive gaming as an avoidance coping mechanism [35, 36, 37], which can lead to a vicious cycle of gaming to relieve distress [38]. For these reasons, it was important to consider the influence of these variables on symptom checklists and behavioral measures in the present study.

### The present study

The primary objective of this study was to evaluate the psychometric properties of the Red Box, Green Box method as a novel self‐report behavioral frequency measure. A focus of this investigation was examining the method's utility for GD screening. The study aimed to test whether Red Box hours (gaming instead of fulfilling responsibilities) had superior diagnostic accuracy and predictive validity for identifying GD compared to the field's conventional approach of total weekly gaming hours. It was hypothesized that Red Box hours would demonstrate a stronger association with GD than Green Box (gaming during free time) hours and total weekly hours. Additionally, the study aimed to identify the optimal cut‐off for Red Box hours to inform its use as a screening tool in research and clinical practice.

## METHODS

### Study design

This prospective, cross‐sectional observational study recruited participants via Prolific, an online crowdsourcing platform that matches participants to study requirements. Participants completed a battery of assessments capturing gaming time, problem gaming symptoms, impulsivity and psychological distress, as well as basic demographic characteristics. All surveys were administered through Qualtrics, an online data collection platform. The study received university ethical approval in June 2024 (ID: HEG7386–5). This study was not pre‐registered, and therefore, analyses should be considered exploratory. A PDF (R Markdown file) document with code used to generate the results is included as a supplementary file. The de‐identified dataset and R script are also publicly available in an online repository [39].

### Participants

Participants were frequent gamers recruited from a pool of 6237 eligible candidates from Australia, Canada, the United States, the United Kingdom and East Asia. Eligibility criteria included: male gender, age 18 to 35 years and self‐reported game exceeding 12 hours weekly. Males were chosen because they are more likely to game highly frequently and report GD, and few GD treatment studies involve females. Participants received standard compensation of 8 GBP per hour through Prolific. A total of 1149 participants met the inclusion criteria and were enrolled into the study.

### Measures

#### Demographic covariates

Participants reported age (in years), nationality, highest education level and current employment status.

#### Gaming time (index measure)

Typical weekly hours were assessed by the item: ‘During the past 6 months, in a typical week, how often do you spend playing video games?’ Respondents are asked to select a number from a drop‐down menu.

#### Red Box, Green Box (index measure)

Green Box hours were measured by asking ‘Can you please estimate the total time (in hours) in a typical week that you spend engaged in gaming during your free time or ‘hobby time’ [i.e. at times for recreation in between your important commitments], or when just passing the time [e.g. when riding the bus]?’ Red Box hours were assessed with the question ‘Can you please estimate the total time (in hours) in a typical week that you spend engaged in gaming at times when you feel that you should be doing something else? This includes gaming when you have other commitments, priorities, or goals or intentions for the day [e.g. studying, working, sleeping, exercising, etc.]?’ From these measures, we calculated total red and green as the sum of Green and Red Box hours, and Red Box proportion as Red Box hours divided by total red and green hours. Both questions were presented sequentially in a fixed order (Green Box, then Red Box) on the same page, with the option to modify responses before proceeding.

#### Problem gaming status (reference measure)

Problem gaming was assessed using the Internet Gaming Disorder Test (IGDT‐10), a 10‐item self‐report questionnaire based on the DSM‐5 criteria IGD [40]. The IGDT‐10 has shown robust psychometric properties in multiple language formats and populations [41]. Two scoring approaches were used to identify potential cases of IGD (i.e. the DSM category) and GD (i.e. the ICD category). In line with DSM guidelines, a cut‐off score of ≥5 indicated IGD. To meet ICD‐11 GD requirements, we used only the five items that referred to loss of control, giving up regular activities, continuation despite harm, risking significant relationships and jeopardizing school/work. Participants who endorsed four or more criteria (including the three essential items, and at least one harm item) were classified with GD. Internal consistency for the IGDT‐10 was ω = 0.77 (internal consistency was assessed using McDonald's ω, which is preferred when α's assumptions are not met) [42].

#### Psychological distress (21‐item Depression, Anxiety and Stress Scale)

The 21‐item Depression, Anxiety and Stress Scale (DASS‐21) [43] assessed psychological distress with seven items each for depression, anxiety and stress. Items were scored on a four‐point Likert scale (0 = never to 3 = almost always). Each subscale ranged from 0 to 21. The DASS‐21 has high internal consistency and cross‐cultural validity [44] for measuring psychological distress, depression and anxiety [45]. Reliability coefficients were ω = 0.93 (depression), ω = 0.85 (anxiety) and ω = 0.89 (stress).

#### Impulsivity (15‐item Barratt Impulsivity Scale)

The 15‐item Barratt Impulsivity Scale (BIS‐15; [46]) assessed overall impulsivity and three impulsivity subtypes: non‐planning, motor and attentional. Responses used a four‐point Likert scale (1 = rarely/never to 4 = almost always), with two reverse‐scored items per subscale. Subscale scores ranged from 5 to 15 (total range: 15–60), with higher scores indicating greater impulsivity. The BIS‐15 has demonstrated good reliability and validity for impulsivity‐related behaviors in healthy individuals [46]. Reliability coefficients were ω = 0.74 (attentional), ω = 0.81 (non‐planning) and ω = 0.82 (motor).

### Statistical analyses

#### Gaming time and problem gaming

Independent samples *t* tests assessed group differences (GD vs. non‐GD) for continuous variables (age, gaming time, psychological distress and impulsivity), while Fisher's exact tests assessed group differences for categorical variables (nationality, employment and education). A zero‐order correlation matrix measured raw associations between variables. A partial‐order correlation matrix assessed relationships between gaming time measures and GD, controlling for demographics, psychological distress and impulsivity.

#### Diagnostic accuracy

Receiver operating characteristics (ROC) curve analyses identified optimal cut‐off scores for identifying problem gaming (using Youden's index) [47]. For GD and IGD cases, we calculated sensitivity as true positive cases divided by total positive cases (TP/[TP + FN]); specificity as true negative cases divided by total negative cases (TN/[TN + FP]); positive predictive value (PPV) as true positives divided by all positive test results (TP/[TP + FP]); and negative predictive value (NPV) as true negatives divided by all negative test results (TN/[TN + FN]). Additionally, we computed the likelihood ratio for positive tests (LR+) as the ratio of true cases to false cases; the likelihood ratio for negative tests (LR−) as the likelihood of true non‐cases to false non‐cases; the clinical utility index for positive tests (CUI+) as the product of sensitivity and PPV; and the clinical utility index for negative tests (CUI−) as the product of specificity and NPV. ROC curves plotted sensitivity against FP (i.e. 1‐specificity), with the criterion for acceptable area under the curve (AUC) set at ≥0.70 [48]. All statistical analyses were conducted using R version 4.4.2 [49] and RStudio version 2024.10.31.

### Sample size justification

A priori power analysis was conducted using the pROC package in R [50] to estimate the minimum required sample size for detecting a difference between two correlated ROC curves. We estimated an AUC value of 0.70 for total weekly gaming hours, which approximates an AUC value considered to have moderate discriminatory value [48]. We assumed a higher AUC of 0.80 for Red Box hours, given the hypothesis that this measure would have higher classification accuracy. A moderate correlation of 0.50 between the two gaming hour predictors was also assumed, and the significance level was set at 0.05 and desired power at 0.90. Results of the analysis indicated that a minimum of 25 cases (i.e. participants meeting criteria for GD) was required. Given the estimated population prevalence of GD of approximately 3% [3], a total sample size of at least 834 participants was required to yield the required number of required cases with paired predictor data.

## RESULTS

### Sample characteristics

Table 1 presents demographic, individual differences and gaming time characteristics. The sample comprised 1149 male participants with a mean age of 25.0 years (SD = 3.5). Most participants were from the United States (53.5%), employed full‐time (39.3%) and/or held college/undergraduate degrees (50.7%). Thirty‐six participants (3.1%) met criteria for GD, while 150 (13.1%) met criteria for IGD (see Table S1). Agreement between ICD and DSM classifications was high, with 34 of 36 participants (94.4%) with GD also classified as having IGD. Notably, the DSM criteria are less stringent and identified more potential cases of IGD than using the ICD approach. The overall mean DASS‐21 score was 20.4 (SD = 14.7), while overall scores for each subscale indicated normal levels of distress [43]. The mean BIS‐15 score was 32.7 (SD = 7.5). When stratified by GD status, *t* tests indicated that participants with GD scored significantly higher on stress, anxiety and depression compared to those without GD (*P* < 0.001). Similarly, participants with GD scored higher on motor and attentional impulsivity than those without GD (*P* < 0.001). No significant differences were observed between groups for age (*P* = 0.800), while Fisher's exact tests indicated no significant differences based on levels of nationality (*P* = 0.710), employment (*P* = 0.502) or education (*P* = 0.494).

**TABLE 1:** Sample characteristics, stratified by ICD‐11 gaming disorder status (*n* = 1149).

Characteristics	Total (*n* = 1149)	Non‐GD (*n* = 1113)	GD (*n* = 36)	*t*	*P*
Age, y; mean (SD)	25 (3.5)	25 (3.5)	24.9 (3.2)	−0.3	0.800
Nationality	–	–	–	N/A	0.713
Australian or New Zealand	46 (4%)	45 (4%)	1 (2.8%)	–	–
American	615 (53.5%)	598 (53.7%)	17 (47.2%)	–	–
European	335 (29.2%)	324 (29.1%)	11 (30.6%)	–	–
Asian	61 (5.3%)	58 (5.2%)	3 (8.3%)	–	–
Other	92 (8%)	88 (7.9%)	4 (11.1%)	–	–
Employment status	–	–	–	N/A	0.503
Casual	49 (4.3%)	46 (4.1%)	3 (8.3%)	–	–
Part‐time	176 (15.3%)	171 (15.4%)	5 (13.9%)	–	–
Full‐time	451 (39.3%)	439 (39.4%)	12 (33.3%)	–	–
Not employed	266 (23.2%)	259 (23.3%)	7 (19.4%)	–	–
Studying	205 (17.8%)	196 (17.6%)	9 (25%)	–	–
Retired	2 (0.2%)	2 (0.2%)	0 (0%)	–	–
Highest educational level attained	–	–	–	N/A	0.485
Secondary/high school	389 (33.9%)	379 (34.1%)	10 (27.8%)	–	–
Further (e.g. apprentice, TAFE)	77 (6.7%)	72 (6.5%)	5 (13.9%)	–	–
Higher (i.e. undergraduate, college)	583 (50.7%)	564 (50.7%)	19 (52.8%)	–	–
Postgraduate (e.g. Masters, PhD)	78 (6.8%)	76 (6.8%)	2 (5.6%)	–	–
Other	21 (1.8%)	21 (1.9%)	0 (0%)	–	–
Gaming time measures	–	–	–	–	–
Red Box hours; mean (SD)	9.1 (8.8)	8.7 (8.4)	21.1 (11.3)	6.5	<0.001
Green Box hours; mean (SD)	22.6 (11.6)	22.4 (11.5)	29 (13.2)	2.9	0.006
Total Green and Red Box hours; mean (SD)	31.7 (17.1)	31.1 (16.7)	50.1 (20.4)	5.5	<0.001
Proportion Red Box hours (%), mean (SD)	27.3 (16.7)	26.8 (16.6)	41.9 (13.6)	6.5	<0.001
Typical weekly hours; mean (SD)	23.7 (6.5)	23.5 (6.5)	27.7 (4.9)	5.0	<0.001
Impulsivity (BIS‐15)	–	–	–	–	–
Non‐planning; mean (SD)	11.9 (3.5)	11.9 (3.5)	13.0 (4.2)	1.6	0.112
Motor; mean (SD)	10.2 (3.2)	10.0 (3.1)	13.8 (4.0)	5.6	<0.001
Attentional; mean (SD)	10.7 (3.0)	10.6 (3.0)	14.0 (3.0)	6.9	<0.001
Total score; mean (SD)	32.7 (7.5)	32.5 (7.3)	40.9 (8.8)	5.7	<0.001
Psychological distress (DASS‐21)	–	–	–	–	–
Stress; mean (SD)	7.4 (5.2)	7.2 (5.2)	13.3 (4.3)	8.4	<0.001
Anxiety; mean (SD)	5.3 (4.7)	5.1 (4.6)	9.8 (5.2)	5.4	<0.001
Depression; mean (SD)	7.7 (6.1)	7.5 (6)	13.6 (5.6)	6.5	<0.001
Total score; mean (SD)	20.4 (14.6)	19.8 (14.4)	36.8 (12.8)	7.8	<0.001

*Note*: GD was classified by endorsement of ≥4 essential ICD‐11 criteria, based on items from IGDT‐10 (see Methods section for details). *t* represents independent samples *t* test statistic assessed group differences for continuous variables. Fisher's exact tests assessed group differences for categorical variables.

Abbreviations: BIS‐15, 15‐item Barratt Impulsivity Scale; DASS‐21, 21‐item Depression, Anxiety and Stress Scale; GD, gaming disorder; ICD‐11, International Classification of Diseases 11th Revision; IGDT‐10, Internet Gaming Disorder Test, 10 item.

### Gaming time

For the typical weekly gaming hours item, participants reported gaming for an average of 23.7 hours (SD = 6.5) per week over the past 6 months. Those with GD reported significantly more typical weekly hours (27.7, SD = 4.9) compared to those without (23.5, SD = 6.5; *t*_39.0_ = 5.0, *P* < 0.001). Red Box and Green Box hours were considered separately and summed together. Participants with GD reported an average of 21.1 Red Box hours (SD = 11.3) and 29.0 Green Box hours (SD = 13.2) per week, which totaled 50.1 hours (SD = 20.4), which was almost twice their reported typical weekly hours. Similarly, participants without GD reported fewer Red Box (8.7, SD = 8.4) and Green Box hours (22.4, SD = 11.5), with total hours (30.1, SD = 3.3) that exceeded their reported typical weekly hours. The proportion of Red Box to total hours (i.e. green plus red) was significantly higher (*t*_38.4_ = 6.5, *P* < 0.001) for those with GD (41.9%, SD = 13.6) compared to those without GD (26.8%, SD = 16.6).

Table 2 presents partial‐order correlations between gaming time measures and GD status, controlling for demographic factors, impulsivity and psychological distress. Typical weekly hours correlated most strongly with Green Box hours (*r* = 0.63, *P* < 0.001), and weakly with GD status (*r* = 0.10, *P* = 0.001). Conversely, Red Box hours showed the strongest correlation with GD status (*r* = 0.19, *P* < 0.001) compared to other measures of gaming time. A zero‐order correlation matrix of all variables appears in Table S2.

**TABLE 2:** Partial‐order correlation matrix for gaming time and problem gaming status (*n* = 1149).

		Typical weekly hours	Red Box hours	Green Box hours	IGDT‐10 items endorsed	GD status	IGD status
1	Typical weekly hours	1.00					
2	Red Box hours	0.31**	1.00				
3	Green Box hours	0.63**	0.37**	1.00			
4	IGDT‐10 items endorsed	0.23**	0.31**	0.19**	1.00		
5	GD status	0.10**	0.19**	0.09*	0.41**	1.00	
6	IGD status	0.15**	0.24**	0.15**	0.76**	0.39**	1.00

Adjusting for age, nationality, education, employment, psychological distress (DASS‐21 total score) and impulsivity (BIS‐15 total score).

Abbreviations: BIS‐15, 15‐item Barratt Impulsivity Scale; DASS‐21, 21‐item Depression, Anxiety and Stress Scale; GD, gaming disorder; ICD‐11, International Classification of Diseases 11th Revision; IGD, internet gaming disorder; IGDT‐10, Internet Gaming Disorder Test, 10 item.

**Significant at *P* < 0.001; *Significant at *P* < 0.05.

### Diagnostic accuracy

**FIGURE 1:**
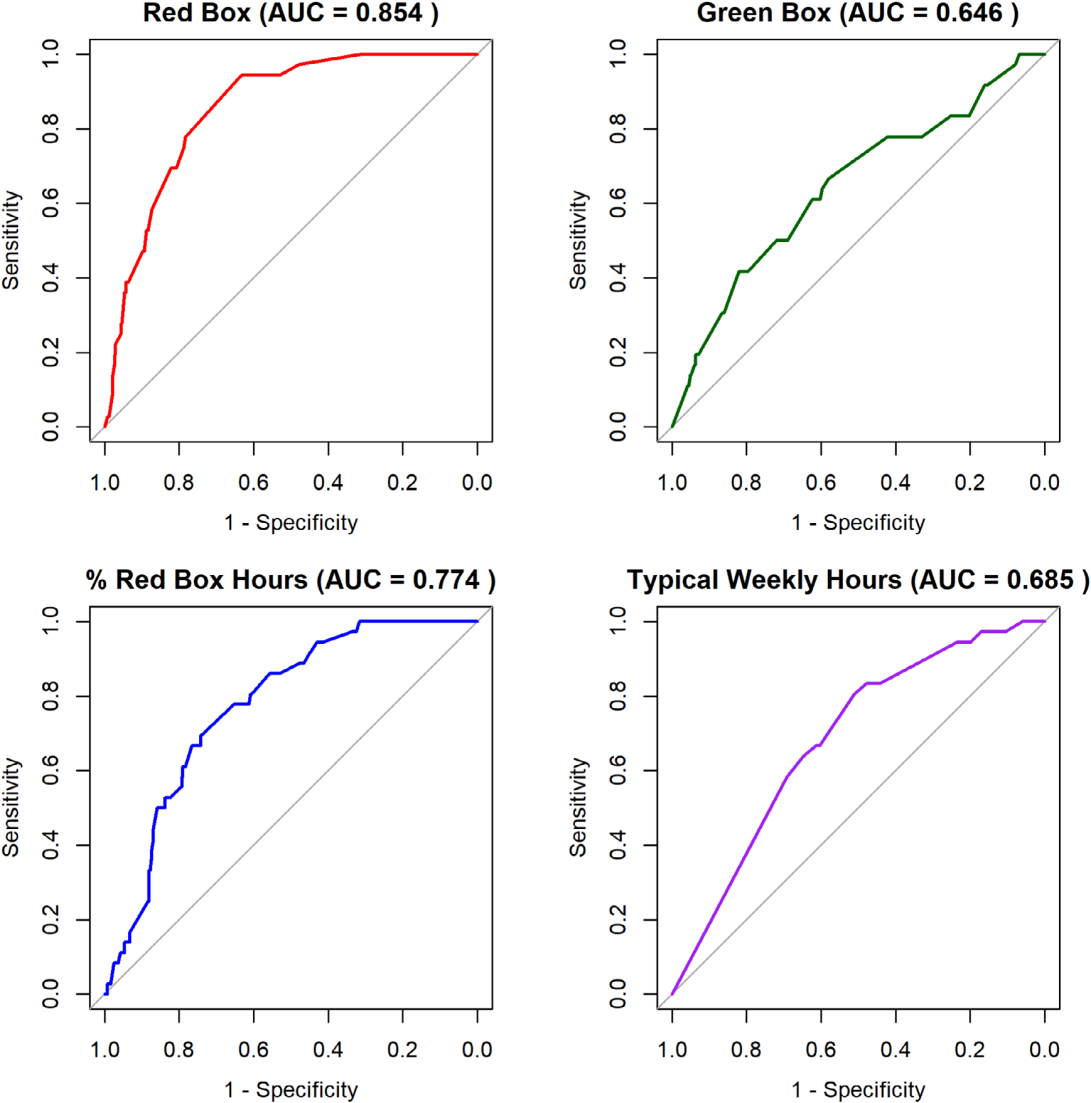
Receiver operating characteristics (ROC) curves plotting gaming time predictor sensitivity as a function of false positive rate for identifying cases of gaming disorder (GD) (International Classification of Diseases 11th Revision). AUC, area under the curve.

Figure 1 displays ROC curves for the four gaming time measures (Red Box hours, Green Box hours, Red Box proportion and typical weekly hours) as predictors of GD, with AUC provided. Red Box hours demonstrated high classification accuracy (AUC = 0.86), followed by Red Box proportion (moderate: AUC = 0.77). Both Green Box hours and typical weekly hours yielded AUCs below the acceptable threshold (>0.70).

Table 3 presents complete diagnostic accuracy indices for gaming time measures as predictors of GD and IGD status. Red Box hours was the superior predictor across multiple indices. For classifying GD, Youden's index identified an optimal cut‐off of 9.5 Red Box hours (AUC = 0.86), yielding a sensitivity of 0.94 and specificity of 0.63. At this threshold, PPV (0.08) and NPV (1.00) were highest among all gaming time measures. The likelihood ratios indicated that individuals with GD were 2.54 times more likely to score above this threshold (LR+), while those below the cut‐off were 10 times less likely to have GD (LR−).

**TABLE 3:** Diagnostic accuracy measures for predicting GD status based on gaming time measures (*n* = 1149).

Predictor	Cut‐off	Sn.	Sp.	PPV	NPV	LR+	LR−	CUI+	CUI−	AUC	OR
ICD‐11 GD											
Red Box (total hours)	9.5	**0.94**	0.63	**0.08**	**1.00**	2.54	0.10	**0.08**	0.63	**0.85**	**29.38**
Green Box (total hours)	20.5	0.67	0.58	0.05	0.98	1.60	**0.57**	0.03	0.57	0.65	2.77
Green + Red Box hours	35.5	0.75	0.70	**0.08**	0.99	2.50	0.36	0.06	0.69	0.78	7.11
Proportion Red Box hours	34.1	0.69	**0.74**	**0.08**	0.99	**2.65**	0.42	0.06	**0.73**	0.77	6.53
Typical weekly hours	24.5	0.81	0.51	0.05	0.99	1.65	0.37	0.04	0.50	0.69	4.35
DSM‐5 IGD											
Red Box (total hours)	5.5	**0.88**	0.56	0.27	**0.96**	2.00	0.21	**0.24**	0.54	**0.76**	**7.83**
Green Box (total hours)	28.5	0.48	0.76	0.26	0.89	2.00	**0.68**	0.12	0.68	0.62	2.63
Green + Red Box hours	38.5	0.58	0.79	**0.33**	0.91	**2.76**	0.53	0.19	**0.72**	0.70	4.36
Proportion Red Box hours	30.9	0.64	0.69	0.27	0.92	2.06	0.52	0.17	0.63	0.69	3.56
Typical weekly hours	24.5	0.71	0.56	0.23	0.92	1.61	0.52	0.16	0.52	0.65	2.86

*Note*: Bold values represent the strongest performing indicator in each of the GD categories. GD was classified by endorsement of ≥4 essential ICD‐11 criteria, based on items from IGDT‐10 (see Methods section for details). DSM‐5 criteria indicated by score of 5+ on IGDT‐10.

Abbreviations: AUC, area under the curve, CUI+, clinical utility index (positive, i.e. case‐finding); CUI−, clinical utility index (negative; screening); DSM‐5, Diagnostic and Statistical Manual of Mental Disorders, Fifth Edition; GD, gaming disorder; ICD‐11, International Classification of Diseases 11th Revision; IGD, internet gaming disorder; IGDT‐10, Internet Gaming Disorder Test, 10 item; LR+, likelihood ratio (positive); LR−, likelihood ratio (negative); NPV, negative predictive value; OR, diagnostic odds ratio; PPV, positive predictive value; Sn., sensitivity; Sp., specificity.

For classifying IGD (the scoring criteria for IGD were less stringent, as a greater number of participants were classed as having IGD, compared to GD), the optimal cut‐off was 5.5 Red Box hours (AUC = 0.76), with sensitivity of 0.88 and specificity of 0.56. NPV at this threshold (0.96) was highest among gaming time indices, while LR+ of 2.00 indicated that individuals with IGD were twice as likely to score above the threshold compared to those without. CUI+ was highest for both DSM‐5 (0.24) and ICD‐11 (0.08) classifications, further supporting Red Box hours' utility as a case‐finding measure compared to other gaming time metrics. Figure S1 provides parallel ROC analyses for predicting IGD status.

## DISCUSSION

This study aimed to assess the psychometric properties of the Red Box, Green Box method as a novel screening approach for aiding identification of problem gaming. A focus of this investigation was the method's utility in screening GD according to ICD‐11 and DSM‐5‐TR criteria. The Red Box, Green Box method was found to be superior to the GD field's conventional total time measure as indicated by its stronger association with GD symptomatology. Red Box hours demonstrated stronger positive associations with GD and IGD compared to Green Box hours and total weekly hours, with good diagnostic accuracy (AUC = 0.86 for GD, AUC = 0.76 for IGD). Notably, the Red Box measure achieved strong psychometric properties (sensitivity of 0.94 for GD) for a single‐item measure, where typically there exists a trade‐off between sensitivity and specificity. However, the measure had a low PPV, which limits its clinical diagnostic value. The strengths of this tool are its brevity and simplicity as a screening tool in epidemiology and general practice.

Another noteworthy observation was that total time measures may underestimate gaming involvement, specifically, total time values tended to closely approximate Green Box values, suggesting that gamers may tend to report their ‘leisure time’ gaming only. This seems comparable to a food intake survey where individuals report food consumption during mealtimes only and fail to report foods (e.g. snacks) consumed at other times of the day. These findings demonstrate the importance of measuring gaming time with reference to context and lifestyle consequences because of gaming, rather than as a singular raw number [7, 25].

The Red Box, Green Box method's psychometric performance suggests that it is a substantial improvement on the conventional total weekly gaming time measure. In King *et al*.’s [8] review of 320 GD studies, the correlation between a GD symptom checklist and self‐report gaming activity tended to fall between 0.25 and 0.50. The present study found that the total gaming time measure was significantly positively correlated with Green Box hours (*r* = 0.64), but less so with Red Box hours (*r* = 0.31). Further, participants with and without GD tended to report Green Box values that were very close to their total time values (i.e. non‐GD group means of 22.6 and 23.7 hours, and GD group means of 22.4 and 23.5 hours, respectively). These results suggest that asking participants to recall gaming time tends to elicit a response based on ‘green time’ (i.e. free time). GD and non‐GD groups appeared quite similar in terms of their gaming patterns when considering only their total gaming time group mean values (i.e. 23.5 vs. 27.7), but GD and non‐GD groups diverged significantly when considering their total time based on the combination of Red and Green Box values (i.e. 31.1 vs. 50.1 hours). These results together indicate that Red Box time (i.e. gaming instead of fulfilling responsibilities) may not be captured well by conventional approaches that measure gaming frequency. Overall, Red Box value was the strongest indicator of GD status. Our analysis of its diagnostic accuracy indicated that reporting 9.5 or more Red Box hours had a 94% likelihood of indicating ICD‐11 GD. Therefore, Red Box time may be a useful single‐item measure for behavioral addiction research and practice, like ‘time to first cigarette’ for nicotine dependence [51, 52].

The ICD‐11 essential criteria for GD refer to increasing priority given to gaming, however, few studies have attempted to measure this criterion using behavioral measures. The present study suggests that problem gamers report, on average, approximately 2.5 times as much gaming activity during ‘red time’ compared to non‐problem gamers. Interestingly, the GD group reported that most of their gaming (~58%, on average) occurred during ‘green time’, which constituted approximately 29 hours per week, which suggests that this group generally viewed their ‘excessive’ gaming as a lesser component of a larger pattern of gaming. These data provide preliminary insights into the dynamic of ‘escalating’ gaming or gaming taking precedence over other interests and activities (i.e. 41% vs. 26% ‘red time’ for GD and non‐GD groups, respectively). These comparisons may inform research agendas with an interest in monitoring the transition of normative to problematic gaming [53]. Longitudinal studies could investigate short‐ and long‐term changes in Red and Green Box values, alongside changes in GD symptomatology and the psychological, social and environmental factors that contribute to shifts in these values.

The Red Box, Green Box approach has practical health applications, from outpatient therapy to digital health platforms, where real‐time monitoring of behavior guides personalized interventions. In clinical practice, the measure could aid in goal‐setting by identifying specific times when gaming is most disruptive (e.g. between 9 am and 5 pm), as GD interventions usually emphasize reducing gaming time [14, 54], with ‘red time’ situations becoming the focus of therapeutic techniques (e.g. emotion regulation strategies, activity scheduling, contingency management) to prevent or reduce problematic use. Post‐therapy, the measure could track client gains by providing context to reductions in gaming time (e.g. emphasizing the client's adaptive use or redirection of gaming to ‘green’ time), rather than aiming for total abstinence [55, 56]. The measure's behavioral focus rather than subjective internal states may be particularly productive for therapy involving parents or family members. Finally, its more concrete or ‘matter of fact’ nature, rather than addiction terminology (e.g. ‘difficulty resisting’ or ‘feeling out of control’), may be better suited to younger clients and clients on the autism spectrum (i.e. individuals over‐represented in problem gaming studies) [57].

The ‘Box’ method may be useful to support broader public health objectives [58, 59]. For education and awareness campaigns, the measure provides a simple language and metric to promote public understanding of problem gaming while avoiding terminology that may elicit negative public reactions, including stigma [60]. The measure could inform the design of consumer tools such as time‐management apps to assist users to balance gaming time with other priorities, as used in controlled‐use approaches in the substance use field [61]. Digital tools with the capability to track Red Box and Green Box dynamics (i.e. linking the user's desired gaming schedule to gaming telemetry data) could identify patterns of behavior that indicate escalating risk of harm (i.e. increasing proportion of ‘red time’ activity), which would trigger notifications and other consumer safety measures.

The present study had limitations. First, the sample included only males age 18 to 35 years who played games regularly, and these findings may not generalize to other populations (e.g. female or adolescent gamers). Although the sample size was adequate for analysis, there was a limited number of participants with GD (i.e. ~3%, which is in line with prevalence studies). There is a need to replicate the study in adolescent, female and clinical populations with more GD cases because gender and age‐related differences in gaming motivation and playing style may potentially affect results [62, 63]. Female gamers are an under‐represented demographic of the gaming population [64] and may require alternative recruitment strategies such as targeting female‐oriented gaming platforms (e.g. mobile gaming, social gaming), partnering with female gaming influencers and engaging gaming communities with higher female participation rates (e.g. simulation, puzzle and social games) [65]. It should be cautioned that the Box method may not yield data that is more accurate than total gaming time measures. Future research should compare Box values with objective tracking information.

Another limitation was the GD screening measure did not evaluate severity of the condition. Similarly, given its brief format, the Red Box does not provide any indication of the degree of interference or seriousness of displacement effects related to gaming (e.g. it does not distinguish between gaming instead of going to work vs. doing laundry, which have different functional consequences). English language proficiency was not assessed. The order of Red versus Green Box questions (and their format; see King *et al*. for options) [10] was not evaluated (all respondents answered the Green and then the Red Box item). Although participants could modify responses before proceeding, it is unclear whether potential double‐counting occurred, (i.e. if they included Red Box time within periods of Green Box time). Similarly, question order may have influenced the individual or combined box values. Further research could examine alternative approaches, such as the use of a sliding scale representing red‐to‐green proportion, or asking participants to first report total gaming time and then divide this value into red and green categories. Crossover study designs could examine these different implementations. Relatedly, future research could clarify how people interpret the categories and delineate red and green time in different scenarios. Although the Box measure is intended to be simple and brief, another potential refinement is an impact item for Red Box time (e.g. see the Patient Health Questionnaire‐9) [66]. Other limitations of the study include lack of specific measurement of awareness or insight into gaming problems, perceptions of gaming‐related stigma and social desirability bias, other mental health conditions and treatment history. Future research could examine whether the Red Box measure is less stigmatizing than standard GD measures and compare the extent to which responses may be affected by social desirability bias.

## CONCLUSIONS

The Red Box, Green Box method offers a simple but promising tool for monitoring gaming behavior and assessing GD risk. These preliminary data indicate that the Box measure is superior in terms of clinical utility to conventional behavioral measurement used in the GD field. The measure's dual focus—which distinguishes gaming as a recreational pursuit from the lifestyle interference caused by gaming—may help to address concerns raised in academic and wider discourse on addiction research lacking sufficient acknowledgement of the relative risks and benefits of gaming. Future work should examine the Box method in different implementations, populations and settings to further evaluate its potential to advance clinical and public health objectives in the field of digital technology‐based addictions.

## AUTHOR CONTRIBUTIONS

**Matthew W. R. Stevens:** Conceptualization (supporting); data curation (lead); formal analysis (lead); investigation (supporting); methodology (equal); software (lead); validation (lead); writing—original draft (equal); writing—review and editing (equal). **Marcela Radünz:** Methodology (supporting); writing—review and editing (supporting). **Christina Galanis:** Methodology (supporting); writing—review and editing (supporting). **Blake Quinney:** Formal analysis (supporting); writing—review and editing (supporting). **Ian Zajac:** Formal analysis (supporting); writing—review and editing (supporting). **Joël Billieux:** Investigation (supporting); methodology (supporting); writing—review and editing (supporting). **Paul H. Delfabbro:** Conceptualization (supporting); formal analysis (supporting); investigation (supporting); methodology (supporting); writing—review and editing (supporting). **Daniel L. King:** Conceptualization (lead); funding acquisition (lead); investigation (equal); methodology (equal); project administration (lead); supervision (lead); writing—original draft (equal); writing—review and editing (supporting).

## DECLARATION OF INTERESTS

The authors report no potential conflicts of interest. The authors alone are responsible for the content and writing of the paper.

## DECLARATION OF GENERATIVE AI IN SCIENTIFIC WRITING

No generative AI was used in this project or this manuscript submission.

## Supporting information


**Data S1.** Supplementary Information.

## Data Availability

A de‐identified dataset and R‐script used to generate the results are available in an online repository [39].
