# Supplementary material for: Red Box, Green Box: Psychometric evaluation of a self‐report behavioral frequency measurement approach for behavioral addictions research
Source: Addiction. 2025 Sep 26;121(2):429–39. doi: 10.1111/add.70192 (PMC12779596; doi:10.1111/add.70192)
Supplement: Supplementary file 1 — Data S1. Supplementary Information. [file ADD-121-429-s001.pdf]

Red Box, Green Box: Psychometric evaluation of a self-report behavioral frequency measurement approach for behavioral addictions research

Statistical Analysis Companion

Matthew W. R. Stevens Marcela Radünz Christina Galanis Blake Quinney
Ian Zajac Joël Billieux Paul H. Delfabbro Daniel L. King

14 August 2025

Contents

INTRODUCTION **2**

DATA PREPARATION, CLEANING AND SETUP **2**

Package Installation . . . 2
Directory Setup (for saving outputs) . . . 2
Data Loading and Inspection . . . 3
Define GD and IGD status . . . 4
Data Structure Validation . . . 5
Data Processing . . . 6
Significance Helper Function . . . 8

RESULTS **8**

Sample size calculation and Power analayis . . . 8
Table 1. Participant Characteristics . . . 9
Table 2. Partial Correlation Matrix . . . 16
Figure 1. ROC Curve Analysis . . . 20
Table 3. Diagnostic Accuracy Indices . . . 26

SUPPLEMENTARY RESULTS **28**

Table S1. Sample Characteristics - By DSM-5 symptom criteria . . . 28
Table S2. Zero Order Correlation Matrix. . . . 29
Figure S1. ROC Curve Analysis (IGD). . . . 32
Contingency Tables (not displayed) . . . 32

REFERENCES **33**

INTRODUCTION

This statistical analysis companion provides complete, comprehensive documentation, and reproducible code for the psychometric evaluation of the "Red Box, Green Box" behavioral frequency measurement approach, published in *Addiction*. Gaming disorder represents a significant public health concern [1], yet the field has historically struggled with accurate measurement of gaming behavior patterns that distinguish between problematic and non-problematic gaming engagement [2,3]. Traditional approaches that rely solely on total gaming hours fail to capture the critical contextual factors that differentiate healthy gaming from pathological gaming behaviors [4,5].

The Red Box, Green Box method represents an innovative re-conceptualization in behavioral frequency assessment, by distinguishing between gaming that occurs during appropriate leisure time (i.e., Green Box hours) versus gaming that replaces essential life responsibilities such as work, education, or self-care (i.e., Red Box hours) [6]. This contextual approach to measurement aligns with contemporary theoretical frameworks of behavioral addictions that emphasize functional impairment and life disruption as core diagnostic features rather than simple quantity metrics.

This document presents the complete analytical framework used to evaluate the psychometric properties, diagnostic accuracy, and clinical utility of this novel measurement approach. The analyses encompass descriptive statistics, correlation matrices, receiver operating characteristic (ROC) curve analyses, and comprehensive diagnostic accuracy indices across both ICD-11 gaming disorder and DSM-5 internet gaming disorder classification systems. The sample includes a large scale statistical evaluation of data from 1,149 male gamers, and demonstrates the superior diagnostic performance of the Red Box metric compared to conventional gaming time mea sures, with implications for clinical screening, epidemiological research, and treatment planning in the behavioral addictions field. The raw (de-identified) data and R code for this analysis are publicly available online [7].

DATA PREPARATION, CLEANING AND SETUP

Package Installation

Check and install required packages for analysis and outputting.

```r
# Package Installation and Loading
required_packages <- c("data.table", "dplyr", "ppcor", "pROC", "kableExtra")

# Check if packages are installed, install if necessary
for (pkg in required_packages) {
  if (!require(pkg, character.only = TRUE, quietly = TRUE)) {
    message(paste("Installing package:", pkg))
    install.packages(pkg, repos = "https://cran.r-project.org")
    library(pkg, character.only = TRUE)
  } else {
    message(paste("Package", pkg, "is already installed and loaded"))
  }
}
```

Directory Setup (for saving outputs)

Set up project directories for saving files.

```r
# Setup Project Directory
setup_project <- function(data_file = "red_box.csv", user_dir = NULL) {
  # Use current directory if no directory is specified
  if (is.null(user_dir)) {
    main_dir <- getwd()
```

```r
    message("Using current working directory: ", main_dir)
  } else {
    if (dir.exists(user_dir)) {
      main_dir <- user_dir
      setwd(main_dir)
      message("Working directory set to: ", main_dir)
    } else {
      stop("Directory does not exist: ", user_dir)
    }
  }

  # Create project structure
  project_dir <- file.path(main_dir, "Green box")
  if (!dir.exists(project_dir)) dir.create(project_dir)

  subdirs <- c("Manuscript Files", "Supplementary Files")
  dir_paths <- list()

  for (subdir in subdirs) {
    path <- file.path(project_dir, subdir)
    if (!dir.exists(path)) dir.create(path)
    # Use consistent naming for list elements
    clean_name <- gsub(" ", "_", tolower(subdir))
    dir_paths[[clean_name]] <- path
  }

  # Check if data file exists in the project directory
  data_path <- file.path(main_dir, data_file)
  if (!file.exists(data_path)) {
    warning(paste("Data file", data_file, "not found in project directory:", data_path))
    warning("Please specify data_file parameter with correct path or place the file in
            the appropriate directory.")
  }

  return(list(
    main_dir = main_dir,
    project_dir = project_dir,
    dir_paths = dir_paths,
    data_path = data_path
  ))
}

project_dirs <- setup_project()
```

Data Loading and Inspection

load and inspect data.

```r
# Data Loading
load_data <- function(data_path) {
  if (!file.exists(data_path)) {
    stop("Data file not found: ", data_path)
  }
```

```
  # Try to load the data
  df <- tryCatch({
    read.csv(data_path, stringsAsFactors = FALSE, na.strings = c("", "NA"))
  }, error = function(e) {
    stop("Error loading data file: ", e$message)
  })

  return(df)
}

# Load the data
df <- load_data(project_dirs$data_path)
```

Define GD and IGD status

The following two functions create columns for classification labelled `GD_status` and `IGD_status`, based on symptoms indicated by the IGDT-10 [8].

For internet gaming disorder (IGD) status, endorsement of at least 5 IGDT-10 criteria indicate the presence of DSM-5 IGD [9].

For gaming disorder (GD) status, a multi-criteria approach was used, whereby endorsement of both essential GD criteria items (item 4: loss of control, and item 6: continued use) was necessary. In addition, endorsement of at least four criteria was necessary from items 4 (impaired control), 5 (i.e., giving up regular activities), 6 (continued use), 9 (risking social relationships) and 10 (risking school or work) [10].

Individuals meeting these criteria were classed as “`GD`” and/or “`IGD`” respectively.

```
# Function to create IGD_status column
create_IGD_status <- function(df) {
  df$IGD_status <- ifelse(df$total_symptoms >= 5, "IGD", "Non-problem")
  return(df)
}

# Function to create GD_status column
create_GD_status <- function(df) {
  # Check if both IGDT_10_4 and IGDT_10_6 equal 1
  condition1 <- (df$IGDT_10_4 == 1) & (df$IGDT_10_6 == 1)

  # Calculate sum of the 5 specified columns
  sum_criteria <- df$IGDT_10_4 + df$IGDT_10_5 + df$IGDT_10_6 + df$IGDT_10_9 + df$IGDT_10_10

  # Check if sum is >= 4
  condition2 <- sum_criteria >= 4

  # Both conditions must be TRUE for GD status
  df$GD_status <- ifelse(condition1 & condition2, "GD", "Non-problem")

  return(df)
}

# Combined function to create both columns at once (optional)
create_both_status <- function(df) {
  df <- create_IGD_status(df)
  df <- create_GD_status(df)
  return(df)
```

```r
}

df <- create_both_status(df)
```

Data Structure Validation

Validate data for required column structure and `GD_status`, `IGD_status`.

Note: Results not displayed here, but use `print(validation_results)` to check output includes number of `GD` and `IGD` cases

```r
# Data Validation
validate_data <- function(df) {
  required_cols <- list(
    numeric = c("age", "red_box", "green_box", "typical_weekly_hours",
                "non_planning", "motor", "attentional",
                "stress", "anxiety", "depression"),
    categorical = c("nationality", "employment", "education"),
    status = c("IGD_status", "GD_status")
  )

  validation_report <- list()

  # Check for required columns
  all_required <- unlist(required_cols)
  missing_cols <- all_required[!all_required %in% colnames(df)]

  if (length(missing_cols) > 0) {
    validation_report$missing_columns <- missing_cols
    warning("Missing required columns: ", paste(missing_cols, collapse = ", "))
  }

  # Check data types for existing columns
  existing_cols <- all_required[all_required %in% colnames(df)]

  for (col in existing_cols) {
    if (col %in% required_cols$numeric) {
      if (!is.numeric(df[[col]])) {
        validation_report$type_issues <- c(validation_report$type_issues,
                                           paste(col, "should be numeric"))
      }
    }
  }

  # Check for minimum sample sizes
  if ("IGD_status" %in% colnames(df)) {
    igd_counts <- table(df$IGD_status, useNA = "ifany")
    validation_report$igd_counts <- igd_counts
  }

  if ("GD_status" %in% colnames(df)) {
    gd_counts <- table(df$GD_status, useNA = "ifany")
    validation_report$gd_counts <- gd_counts
  }
```

```r
  return(validation_report)
}

validation_results <- validate_data(df)
```

Data Processing

Define function to preprocess data. This involves ensuring variables are either numeric, or factors (if categorical), creating binary classification variables for later ROC curve analysis, and creating red and green hours, and red box proportion.

```r
# Data Processing
process_data <- function(df) {
  # Create a copy to avoid modifying original
  df_processed <- df

  # Convert categorical variables to factors and create numeric versions
  categorical_vars <- c("nationality", "employment", "education")

  for (var_name in categorical_vars) {
    if (var_name %in% colnames(df_processed)) {
      df_processed[[var_name]] <- as.factor(df_processed[[var_name]])
      # Create numeric recoded version
      recode_name <- paste0(var_name, "_recoded")
      df_processed[[recode_name]] <- as.numeric(df_processed[[var_name]])
    } else {
      warning(paste("Categorical variable", var_name, "not found in data"))
      # Create dummy variables
      df_processed[[var_name]] <- factor("Unknown")
      df_processed[[paste0(var_name, "_recoded")]] <- 1
    }
  }

  # Create binary status variables
  if ("IGD_status" %in% colnames(df_processed)) {
    df_processed$IGD_status <- as.factor(df_processed$IGD_status)
    df_processed$IGD_status_binary <- as.numeric(df_processed$IGD_status == "IGD")
  } else {
    warning("IGD_status not found, creating dummy variable")
    df_processed$IGD_status <- factor("Non-IGD")
    df_processed$IGD_status_binary <- 0
  }

  if ("GD_status" %in% colnames(df_processed)) {
    df_processed$GD_status <- as.factor(df_processed$GD_status)
    df_processed$GD_status_binary <- as.numeric(df_processed$GD_status == "GD")
  } else {
    warning("GD_status not found, creating dummy variable")
    df_processed$GD_status <- factor("Non-GD")
    df_processed$GD_status_binary <- 0
  }

  # Calculate combined variables
  if (all(c("green_box", "red_box") %in% colnames(df_processed))) {
```

```r
    df_processed$green_plus_red <- df_processed$green_box + df_processed$red_box
    df_processed$red_proportion <- (df_processed$red_box / df_processed$green_plus_red) * 100
    # Handle division by zero
    df_processed$red_proportion[is.infinite(df_processed$red_proportion)] <- 0
  } else {
    warning("green_box or red_box not found, cannot calculate combined variables")
    df_processed$green_plus_red <- 0
    df_processed$red_proportion <- 0
  }

  # Create total_symptoms if it doesn't exist
  if (!"total_symptoms" %in% colnames(df_processed)) {
    # Assume it's sum of some symptom measures - adjust as needed
    symptom_cols <- c("stress", "anxiety", "depression")
    existing_symptom_cols <- symptom_cols[symptom_cols %in% colnames(df_processed)]

    if (length(existing_symptom_cols) > 0) {
      df_processed$total_symptoms <- rowSums(df_processed[existing_symptom_cols], na.rm = TRUE)
    } else {
      df_processed$total_symptoms <- 0
      warning("No symptom columns found, setting total_symptoms to 0")
    }
  }

  # Create total impulsivity if it doesn't exist
  if (!"total_impulsivity" %in% colnames(df_processed)) {
    impulsivity_cols <- c("non_planning", "motor", "attentional")
    existing_imp_cols <- impulsivity_cols[impulsivity_cols %in% colnames(df_processed)]

    if (length(existing_imp_cols) > 0) {
      df_processed$total_impulsivity <- rowSums(df_processed[existing_imp_cols], na.rm = TRUE)
    } else {
      df_processed$total_impulsivity <- 0
      warning("No impulsivity columns found, setting total_impulsivity to 0")
    }
  }

  # Create DASS total if it doesn't exist
  if (!"DASS_total" %in% colnames(df_processed)) {
    dass_cols <- c("stress", "anxiety", "depression")
    existing_dass_cols <- dass_cols[dass_cols %in% colnames(df_processed)]

    if (length(existing_dass_cols) > 0) {
      df_processed$DASS_total <- rowSums(df_processed[existing_dass_cols], na.rm = TRUE)
    } else {
      df_processed$DASS_total <- 0
      warning("No DASS columns found, setting DASS_total to 0")
    }
  }

  return(df_processed)
}

df <- process_data(df)
```

Significance Helper Function

Define helper functions to add significance indicators to correlation tables.

```
# Helper Functions
get_sig <- function(p) {
  if (is.na(p)) return("")
  if (p < 0.001) return("**")
  else if (p < 0.05) return("*")
  else return("")
}

get_sig_fdr <- function(p_adj) {
  if (is.na(p_adj)) return("")
  if (p_adj < 0.001) return("***")
  if (p_adj < 0.01) return("**")
  if (p_adj < 0.05) return("*")
  return("")
}
```

RESULTS

Sample size calculation and Power analayis

Use pROC package [11] to calculate required sample size. Calculations based on predicted AUC of 0.80, compared to a null AUC of 0.7, assuming correlation between the two of 0.50, setting type-I error rate to 0.05, and assuming a prevalence rate of 3% [12, 13].

```
# Inputs
auc1 <- 0.80 # AUC of better predictor
auc2 <- 0.70 # AUC of comparison predictor
correlation <- 0.50 # Correlation between predictors
power_target <- 0.90 # Desired power
alpha <- 0.05 # Significance level
prevalence <- 0.03 # Estimated prevalence of Gaming Disorder

# Run power analysis
power_result <- power.roc.test(
  auc = auc1,
  auc2 = auc2,
  power = power_target,
  sig.level = alpha,
  kappa = correlation)

# Extract required number of cases
n_cases <- ceiling(power_result$ncases)

# Compute total sample size required to yield that many cases
total_sample <- ceiling(n_cases / prevalence)

# Display results as a formatted table
pROCresults <- data.frame(
  Metric = c("Required Cases", "Estimated Total Sample", "Estimated Diagnostic Prevalence"),
  Value = c(
```

Sample Size Calculations for ROC Curve Analysis

Metric	Value
Required Cases	25
Estimated Total Sample	834
Estimated Diagnostic Prevalence	3%

```
    format(n_cases, big.mark = ","),
    format(total_sample, big.mark = ","),
    paste0(prevalence * 100, "%")
  )
)

# Create nicely formatted table
kbl(pROCresults,
      col.names = c("Metric", "Value"),
      align = c("l", "r"),
      caption = "Sample Size Calculations for ROC Curve Analysis") %>%
  kable_styling(
    bootstrap_options = c("striped", "hover", "condensed"),
    full_width = FALSE,
    position = "left"
  ) %>%
  column_spec(1, bold = FALSE, width = "5cm") %>%
  column_spec(2, width = "3cm")
```

Table 1. Participant Characteristics

The following function creates table 1 of the manuscript, which outlines participant characteristics stratified by GD or non-GD groups, and includes t- and chi-square tests of group differences.

```
# Descriptive Statistics Functions
create_descriptive_table <- function(df, group_var = "GD_status", group_value = "GD",
                                     table_title = "Sample Characteristics by GD Status") {
  # Calculate total sample size and group sizes
  total_n <- nrow(df)
  non_problem_n <- sum(df[[group_var]] != group_value, na.rm = TRUE)
  problem_n <- sum(df[[group_var]] == group_value, na.rm = TRUE)

  # Function to format means and SDs
  format_mean_sd <- function(x) {
    mean_val <- round(mean(x, na.rm = TRUE), 1)
    sd_val <- format(round(sd(x, na.rm = TRUE), 1), nsmall=1)
    return(paste0(mean_val, " (", sd_val, ")"))
  }

  # Function to format counts and percentages
  format_count_pct <- function(x, n) {
    count <- sum(x, na.rm = TRUE)
    pct <- format(round(count/n * 100, 1), nsmall = 1)
    return(paste0(count, " (", pct, "%", ")"))
  }
```

```r
# Function to perform t-test between groups
perform_ttest <- function(x, group) {
  if(sum(!is.na(x[group == group_value])) < 2 || sum(!is.na(x[group != group_value])) < 2) {
    return(list(t_value = "N/A", p_value = "N/A"))
  }

  t_result <- tryCatch({
    test <- t.test(x ~ group)
    list(
      t_value = format(round(test$statistic, 1), nsmall =1),
      p_value = if(test$p.value < 0.001) "<.001" else format(round(test$p.value, 3), nsmall=3)
    )
  }, error = function(e) {
    list(t_value = "N/A", p_value = "N/A")
  })

  return(t_result)
}

# Function to perform chi-square test for categorical variables
perform_chisq <- function(x, group) {
  tab <- table(x, group)
  if(any(tab < 5)) {
    # Use Fisher's exact test for small sample sizes
    test <- tryCatch({
      fisher.test(tab)
    }, error = function(e) {
      return(list(p.value = NA))
    })
  } else {
    test <- tryCatch({
      chisq.test(tab)
    }, error = function(e) {
      return(list(p.value = NA))
    })
  }

  p_value <- if(is.na(test$p.value)) {
    "N/A"
  } else if(test$p.value < 0.001) {
    "<.001"
  } else {
    format(round(test$p.value, 3), nsmall=3)
  }

  return(list(t_value = "N/A", p_value = p_value))
}

# Initialize the results table
table_data <- data.frame(
  Characteristic = character(),
  Total = character(),
  NonProblem = character(),
  Problem = character(),
  t = character(),
```

```r
  p = character(),
  stringsAsFactors = FALSE
)

# Add age row
age_ttest <- perform_ttest(df$age, df[[group_var]])
table_data <- rbind(table_data, data.frame(
  Characteristic = "Age, years; mean (SD)",
  Total = format_mean_sd(df$age),
  NonProblem = format_mean_sd(df$age[df[[group_var]] != group_value]),
  Problem = format_mean_sd(df$age[df[[group_var]] == group_value]),
  t = age_ttest$t_value,
  p = age_ttest$p_value,
  stringsAsFactors = FALSE
))

# Add nationality
nationality_test <- perform_chisq(df$nationality, df[[group_var]])
table_data <- rbind(table_data, data.frame(
  Characteristic = "Nationality",
  Total = "--",
  NonProblem = "--",
  Problem = "--",
  t = "N/A",
  p = nationality_test$p_value,
  stringsAsFactors = FALSE
))

# Get all unique nationalities and add them as subrows
nationalities <- sort(unique(df$nationality))
for(nat in nationalities) {
  if(!is.na(nat) && nat != "") {
    is_nat <- df$nationality == nat
    table_data <- rbind(table_data, data.frame(
      Characteristic = paste("  ", nat),
      Total = format_count_pct(is_nat, total_n),
      NonProblem = format_count_pct(is_nat & df[[group_var]] != group_value, non_problem_n),
      Problem = format_count_pct(is_nat & df[[group_var]] == group_value, problem_n),
      t = "-",
      p = "-",
      stringsAsFactors = FALSE
    ))
  }
}

# Add employment status
employment_test <- perform_chisq(df$employment, df[[group_var]])
table_data <- rbind(table_data, data.frame(
  Characteristic = "Employment status",
  Total = "--",
  NonProblem = "--",
  Problem = "--",
  t = "N/A",
  p = employment_test$p_value,
  stringsAsFactors = FALSE
```

```
))

# Get all unique employment statuses and add them as subrows
employment_statuses <- sort(unique(df$employment))
for(emp in employment_statuses) {
  if(!is.na(emp) && emp != "") {
    is_emp <- df$employment == emp
    table_data <- rbind(table_data, data.frame(
      Characteristic = paste("  ", emp),
      Total = format_count_pct(is_emp, total_n),
      NonProblem = format_count_pct(is_emp & df[[group_var]] != group_value, non_problem_n),
      Problem = format_count_pct(is_emp & df[[group_var]] == group_value, problem_n),
      t = "-",
      p = "-",
      stringsAsFactors = FALSE
    ))
  }
}

# Add education
education_test <- perform_chisq(df$education, df[[group_var]])
table_data <- rbind(table_data, data.frame(
  Characteristic = "Highest educational level attained",
  Total = "--",
  NonProblem = "--",
  Problem = "--",
  t = "N/A",
  p = education_test$p_value,
  stringsAsFactors = FALSE
))

# Get all unique education levels and add them as subrows
education_levels <- sort(unique(df$education))
for(edu in education_levels) {
  if(!is.na(edu) && edu != "") {
    is_edu <- df$education == edu
    table_data <- rbind(table_data, data.frame(
      Characteristic = paste("  ", edu),
      Total = format_count_pct(is_edu, total_n),
      NonProblem = format_count_pct(is_edu & df[[group_var]] != group_value, non_problem_n),
      Problem = format_count_pct(is_edu & df[[group_var]] == group_value, problem_n),
      t = "-",
      p = "-",
      stringsAsFactors = FALSE
    ))
  }
}

# Add gaming time measures header
table_data <- rbind(table_data, data.frame(
  Characteristic = "Gaming time measures",
  Total = "--",
  NonProblem = "--",
  Problem = "--",
  t = "-",
```

```r
  p = "-",
  stringsAsFactors = FALSE
))

# Add gaming time metrics
gaming_metrics <- list(
  list(name = "Red box hours; mean (SD)", var = "red_box"),
  list(name = "Green box hours; mean (SD)", var = "green_box"),
  list(name = "Total green & red hours; mean (SD)", var = "green_plus_red"),
  list(name = "Proportion red box hours (%), mean (SD)", var = "red_proportion"),
  list(name = "Typical weekly hours; mean (SD)", var = "typical_weekly_hours")
)

for(metric in gaming_metrics) {
  t_test <- perform_ttest(df[[metric$var]], df[[group_var]])
  table_data <- rbind(table_data, data.frame(
    Characteristic = metric$name,
    Total = format_mean_sd(df[[metric$var]]),
    NonProblem = format_mean_sd(df[[metric$var]][df[[group_var]] != group_value]),
    Problem = format_mean_sd(df[[metric$var]][df[[group_var]] == group_value]),
    t = t_test$t_value,
    p = t_test$p_value,
    stringsAsFactors = FALSE
  ))
}

# Add impulsivity header
table_data <- rbind(table_data, data.frame(
  Characteristic = "Impulsivity (BIS-15)",
  Total = "--",
  NonProblem = "--",
  Problem = "--",
  t = "-",
  p = "-",
  stringsAsFactors = FALSE
))

# Add impulsivity metrics
impulsivity_metrics <- list(
  list(name = "Non-planning; mean (SD)", var = "non_planning"),
  list(name = "Motor; mean (SD)", var = "motor"),
  list(name = "Attentional; mean (SD)", var = "attentional"),
  list(name = "Total score; mean (SD)", var = "total_impulsivity")
)

for(metric in impulsivity_metrics) {
  t_test <- perform_ttest(df[[metric$var]], df[[group_var]])
  table_data <- rbind(table_data, data.frame(
    Characteristic = metric$name,
    Total = format_mean_sd(df[[metric$var]]),
    NonProblem = format_mean_sd(df[[metric$var]][df[[group_var]] != group_value]),
    Problem = format_mean_sd(df[[metric$var]][df[[group_var]] == group_value]),
    t = t_test$t_value,
    p = t_test$p_value,
    stringsAsFactors = FALSE
```

```r
    ))
  }

  # Add psychological distress header
  table_data <- rbind(table_data, data.frame(
    Characteristic = "Psychological distress (DASS-21)",
    Total = "--",
    NonProblem = "--",
    Problem = "--",
    t = "-",
    p = "-",
    stringsAsFactors = FALSE
  ))

  # Add psychological distress metrics
  distress_metrics <- list(
    list(name = "Stress; mean (SD)", var = "stress"),
    list(name = "Anxiety; mean (SD)", var = "anxiety"),
    list(name = "Depression; mean (SD)", var = "depression"),
    list(name = "Total score; mean (SD)", var = "DASS_total")
  )

  for(metric in distress_metrics) {
    t_test <- perform_ttest(df[[metric$var]], df[[group_var]])
    table_data <- rbind(table_data, data.frame(
      Characteristic = metric$name,
      Total = format_mean_sd(df[[metric$var]]),
      NonProblem = format_mean_sd(df[[metric$var]][df[[group_var]] != group_value]),
      Problem = format_mean_sd(df[[metric$var]][df[[group_var]] == group_value]),
      t = t_test$t_value,
      p = t_test$p_value,
      stringsAsFactors = FALSE
    ))
  }

  # Rename columns to match the desired format
  colnames(table_data) <- c(
    "Characteristics",
    paste0("Total (N=", total_n, ")"),
    paste0("Non-problem (N=", non_problem_n, ")"),
    paste0(group_value, " (N=", problem_n, ")"),
    "t",
    "p"
  )

  return(table_data)
}
```

Call the function and print the table.

```r
# Call function and save object
table_1 <- create_descriptive_table(df, group_var = "GD_status", group_value = "GD")

# Save tables
write.csv(table_1,
```

```r
          file.path(project_dirs$dir_paths$manuscript_files,
                    "Table 1 - Sample Characteristics.csv"),
          row.names = FALSE)

footnote <- "Gaming disorder (GD) was classified by endorsement of 4 or more essential ICD-11 criteria, bas

kbl(table_1,
    caption = "Table 1. Sample characteristics, stratified by ICD-11 gaming disorder
    status (N=1,149)",
    col.names = c("Characteristics", "Total (N=1,149)", "Non-GD (N=1,113)", "GD (N=36)",
                  "t/X2", "p"),
    booktabs = TRUE,
    longtable = TRUE,
    row.names = FALSE,
    escape = TRUE,
    align = c("l", "r", "r", "r", "r", "r")) %>%
  kable_styling(latex_options = c("striped", "hover", "condensed", "responsive"),
                font_size = 10) %>%
  column_spec(1, width = "6.4cm") %>%
  column_spec(2:6, width_min = "1.2cm", width_max = "3.0cm") %>%
  footnote(general = footnote,
           threeparttable = TRUE,
           escape = TRUE)
```

Table 1. Sample characteristics, stratified by ICD-11 gaming disorder status (N=1,149)

Characteristics	Total (N=1,149)	Non-GD (N=1,113)	GD (N=36)	t/X2	p
Age, years; mean (SD)	25 (3.5)	25 (3.5)	24.9 (3.2)	-0.3	0.800
Nationality	–	–	–	N/A	0.713
American	615 (53.5%)	598 (53.7%)	17 (47.2%)	-	-
Asian	61 (5.3%)	58 (5.2%)	3 (8.3%)	-	-
Australian or New Zealand	46 (4.0%)	45 (4.0%)	1 (2.8%)	-	-
European	335 (29.2%)	324 (29.1%)	11 (30.6%)	-	-
Other	92 (8.0%)	88 (7.9%)	4 (11.1%)	-	-
Employment status	–	–	–	N/A	0.503
Casual	49 (4.3%)	46 (4.1%)	3 (8.3%)	-	-
Full-time	451 (39.3%)	439 (39.4%)	12 (33.3%)	-	-
Not Employed	266 (23.2%)	259 (23.3%)	7 (19.4%)	-	-
Part-time	176 (15.3%)	171 (15.4%)	5 (13.9%)	-	-
Retired	2 (0.2%)	2 (0.2%)	0 (0.0%)	-	-
Studying	205 (17.8%)	196 (17.6%)	9 (25.0%)	-	-
Highest educational level attained	–	–	–	N/A	0.501
#NULL!	1 (0.1%)	1 (0.1%)	0 (0.0%)	-	-
Further (e.g. Apprentice, TAFE)	77 (6.7%)	72 (6.5%)	5 (13.9%)	-	-
Higher (ie. Undergraduate, College)	583 (50.7%)	564 (50.7%)	19 (52.8%)	-	-
Other (please Specify)	21 (1.8%)	21 (1.9%)	0 (0.0%)	-	-
Postgraduate (e.g. Masters, Ph.D)	78 (6.8%)	76 (6.8%)	2 (5.6%)	-	-
Secondary/High School	389 (33.9%)	379 (34.1%)	10 (27.8%)	-	-
Gaming time measures	–	–	–	-	-
Red box hours; mean (SD)	9.1 (8.8)	8.7 (8.4)	21.1 (11.3)	6.5	<.001
Green box hours; mean (SD)	22.6 (11.6)	22.4 (11.5)	29 (13.2)	2.9	0.006
Total green & red hours; mean (SD)	31.7 (17.1)	31.1 (16.7)	50.1 (20.4)	5.5	<.001

Proportion red box hours (%), mean (SD)	27.3 (16.7)	26.8 (16.6)	41.9 (13.6)	6.5	<.001
Typical weekly hours; mean (SD)	23.7 (6.5)	23.5 (6.5)	27.7 (4.9)	5.0	<.001
Impulsivity (BIS-15)	–	–	–	-	-
Non-planning; mean (SD)	11.9 (3.5)	11.9 (3.5)	13 (4.2)	1.6	0.112
Motor; mean (SD)	10.2 (3.2)	10 (3.1)	13.8 (4.0)	5.6	<.001
Attentional; mean (SD)	10.7 (3.0)	10.6 (3.0)	14 (3.0)	6.9	<.001
Total score; mean (SD)	32.7 (7.5)	32.5 (7.3)	40.9 (8.8)	5.7	<.001
Psychological distress (DASS-21)	–	–	–	-	-
Stress; mean (SD)	7.4 (5.2)	7.2 (5.2)	13.3 (4.3)	8.4	<.001
Anxiety; mean (SD)	5.3 (4.7)	5.1 (4.6)	9.8 (5.2)	5.4	<.001
Depression; mean (SD)	7.7 (6.1)	7.5 (6.0)	13.6 (5.6)	6.5	<.001
Total score; mean (SD)	20.4 (14.6)	19.8 (14.4)	36.8 (12.8)	7.8	<.001

Note:
Gaming disorder (GD) was classified by endorsement of 4 or more essential ICD-11 criteria, based on items from IGDT-10. BIS-15 = 15-item Barratt Impulsivity Scale. DASS-21 = 21-item Depression, Anxiety and Stress Scale.

Table 2. Partial Correlation Matrix

The following functions creates the partial and zero-order correlation matrices separately.

```r
# Correlation Analysis Functions
partial_cor_matrix <- function(df_complete) {
  vars <- c("typical_weekly_hours", "red_box", "green_box", "total_symptoms",
            "GD_status_binary", "IGD_status_binary")
  control_vars <- c("non_planning", "attentional", "motor", "stress",
                    "anxiety", "depression", "nationality_recoded",
                    "education_recoded", "employment_recoded")

  # Check which variables exist
  existing_vars <- vars[vars %in% colnames(df_complete)]
  existing_controls <- control_vars[control_vars %in% colnames(df_complete)]

  if (length(existing_vars) < 2) {
    warning("Not enough variables for correlation analysis")
    return(matrix("N/A", nrow = length(vars), ncol = length(vars),
                  dimnames = list(vars, vars)))
  }

  cor_matrix <- matrix(NA, nrow = length(existing_vars), ncol = length(existing_vars),
                       dimnames = list(existing_vars, existing_vars))
  p_matrix <- matrix(NA, nrow = length(existing_vars), ncol = length(existing_vars),
                     dimnames = list(existing_vars, existing_vars))

  diag(cor_matrix) <- 1

  # Store all p-values for FDR correction
  all_p_values <- c()
  p_value_positions <- list()

  for (i in 1:(length(existing_vars) - 1)) {
    for (j in (i + 1):length(existing_vars)) {
      tryCatch({
```

```r
      if (length(existing_controls) > 0) {
        test <- ppcor::pcor.test(df_complete[[existing_vars[i]]],
                                 df_complete[[existing_vars[j]]],
                                 df_complete[, existing_controls])
      } else {
        # Fall back to regular correlation if no control variables
        test <- cor.test(df_complete[[existing_vars[i]]],
                         df_complete[[existing_vars[j]]])
      }

      cor_val <- test$estimate
      p_val <- test$p.value

      cor_matrix[existing_vars[i], existing_vars[j]] <- cor_val
      cor_matrix[existing_vars[j], existing_vars[i]] <- cor_val
      p_matrix[existing_vars[i], existing_vars[j]] <- p_val
      p_matrix[existing_vars[j], existing_vars[i]] <- p_val

      # Store p-values for FDR correction
      if (!is.na(p_val)) {
        all_p_values <- c(all_p_values, p_val)
        p_value_positions[[length(all_p_values)]] <- c(i, j)
      }

    }, error = function(e) {
      warning(paste("Error calculating correlation between",
                    existing_vars[i], "and", existing_vars[j], ":", e$message))
    })
  }
}

# Apply Benjamini-Hochberg FDR correction
if (length(all_p_values) > 0) {
  adjusted_p_values <- p.adjust(all_p_values, method = "BH")

  # Create adjusted p-value matrix
  p_matrix_adj <- matrix(NA, nrow = length(existing_vars), ncol = length(existing_vars),
                         dimnames = list(existing_vars, existing_vars))

  # Fill in adjusted p-values
  for (k in 1:length(adjusted_p_values)) {
    pos <- p_value_positions[[k]]
    i <- pos[1]
    j <- pos[2]
    p_matrix_adj[existing_vars[i], existing_vars[j]] <- adjusted_p_values[k]
    p_matrix_adj[existing_vars[j], existing_vars[i]] <- adjusted_p_values[k]
  }
} else {
  p_matrix_adj <- p_matrix
}

# Create character matrix with FDR-corrected significance markers
char_matrix <- matrix("", nrow = length(existing_vars), ncol = length(existing_vars),
                      dimnames = list(existing_vars, existing_vars))
for (i in 1:length(existing_vars)) {
```

```
    for (j in 1:length(existing_vars)) {
      if (i == j) {
        char_matrix[i, j] <- "1.00"
      } else {
        if (!is.na(cor_matrix[i, j])) {
          cor_val <- sprintf("%.2f", cor_matrix[i, j])
          # Use FDR-adjusted p-values for significance markers
          sig <- get_sig_fdr(p_matrix_adj[i, j])
          char_matrix[i, j] <- paste0(cor_val, sig)
        } else {
          char_matrix[i, j] <- "N/A"
        }
      }
    }
  }

  return(char_matrix)

}
```

```
# Zero-Order Correlation Matrix Function
zero_order_cor_matrix <- function(df_complete) {
  vars <- c("typical_weekly_hours", "red_box", "green_box", "total_symptoms",
            "GD_status_binary", "IGD_status_binary", "non_planning",
            "attentional", "motor", "stress", "anxiety", "depression",
            "age", "nationality_recoded", "employment_recoded", "education_recoded")

  cor_matrix_2 <- cor(df_complete[, vars], use = "pairwise.complete.obs")

  p_matrix_2 <- matrix(NA, nrow = length(vars), ncol = length(vars),
                       dimnames = list(vars, vars))

  for (i in 1:(length(vars) - 1)) {
    for (j in (i + 1):length(vars)) {
      tryCatch({
        test <- cor.test(df_complete[[vars[i]]], df_complete[[vars[j]]])
        p_val <- test$p.value
        # Fixed: Changed p_matrix to p_matrix_2
        p_matrix_2[vars[i], vars[j]] <- p_val
        p_matrix_2[vars[j], vars[i]] <- p_val
      }, error = function(e) {
        warning(paste("Error calculating correlation between",
                      vars[i], "and", vars[j], ":", e$message))
      })
    }
  }

  char_matrix_2 <- matrix("", nrow = length(vars), ncol = length(vars),
                          dimnames = list(vars, vars))
  for (i in 1:length(vars)) {
    for (j in 1:length(vars)) {
      if (i == j) {
        char_matrix_2[i, j] <- "1.00"
      } else {
        cor_val <- sprintf("%.2f", cor_matrix_2[i, j])
```

```
        sig <- get_sig(p_matrix_2[i, j])
        char_matrix_2[i, j] <- paste0(cor_val, sig)
      }
    }
  }

  return(char_matrix_2)
}
```

Construct both matrices, and save them into the manuscript and supplementary directories.

```
# Define required variables for correlation analysis
required_vars_for_analysis <- c(
  "typical_weekly_hours", "red_box", "green_box", "IGD_status_binary",
  "GD_status_binary", "total_symptoms", "non_planning", "attentional",
  "motor", "total_impulsivity", "stress", "anxiety", "depression",
  "DASS_total", "age", "nationality_recoded", "employment_recoded",
  "education_recoded"
)

# Only use variables that actually exist in the dataset
existing_analysis_vars <- required_vars_for_analysis[required_vars_for_analysis
                                                    %in% colnames(df)]

df_complete <- df[complete.cases(df[, existing_analysis_vars]), ]

# Compute correlation matrices
partial_cor_mat <- partial_cor_matrix(df_complete)

# Save partial correlation matrix
write.csv(partial_cor_mat,
          file.path(project_dirs$dir_paths$manuscript_files,
                    "Table 2 - Partial-order correlation matrix.csv"),
          row.names = TRUE)
```

Print the partial order matrix.

```
# Create upper triangular matrix for publication table
upper_triangle_matrix <- partial_cor_mat
upper_triangle_matrix[lower.tri(upper_triangle_matrix, diag = FALSE)] <- ""

# Convert to data frame
corr_df <- as.data.frame(upper_triangle_matrix)

new_names <- c(
  "Typical weekly hours",
  "Red Box hours",
  "Green Box hours",
  "IGDT-10 items endorsed",
  "GD status",
  "IGD status"
)

# Apply new row names
if(nrow(corr_df) <= length(new_names)) {
```

```
  rownames(corr_df) <- new_names[1:nrow(corr_df)]
}

# Create table
correlation_table <- kbl(corr_df,
                        caption = "Table 2. Partial-order correlation matrix for
                        gaming time and problem gaming status",
                        booktabs = TRUE,
                        longtable = TRUE,
                        col.names = if(ncol(corr_df) <= length(new_names)) {
                          new_names[1:ncol(corr_df)]
                        } else {
                          c(new_names, colnames(corr_df)[(length(new_names)+1):ncol(corr_df)])
                        }) %>%
  kable_styling(latex_options = c("striped", "hold_position", "repeat_header"),
                font_size = 10) %>%
  column_spec(1, width = "6cm") %>%
  column_spec(2:7, width = "1.5cm") %>%
  footnote(general = "Adjusting for age, nationality, education, employment,
          psychological distress, and impulsivity, *p<0.05; **p<0.01; **p<.001",
          threeparttable = TRUE,
          escape = FALSE)

correlation_table
```

Table 2. Partial-order correlation matrix for gaming time and problem gaming status

	Typical weekly hours	Red Box hours	Green Box hours	IGDT-10 items endorsed	GD status	IGD status
Typical weekly hours	1.00	0.31***	0.63***	0.23***	0.10***	0.15***
Red Box hours		1.00	0.37***	0.31***	0.19***	0.24***
Green Box hours			1.00	0.19***	0.09**	0.15***
IGDT-10 items endorsed				1.00	0.41***	0.76***
GD status					1.00	0.39***
IGD status						1.00

Note:
Adjusting for age, nationality, education, employment, psychological distress, and impulsivity, *p<0.05; **p<0.01; **p<.001

Figure 1. ROC Curve Analysis

This code generates Figure 1 (ROC curves) and Table 3 (diagnostic accuracy indices)

```
# Compute diagnostic accuracy indices
calc_all_indices <- function(pred, ref) {
  TP <- sum(pred == 1 & ref == 1, na.rm = TRUE)
  TN <- sum(pred == 0 & ref == 0, na.rm = TRUE)
  FP <- sum(pred == 1 & ref == 0, na.rm = TRUE)
  FN <- sum(pred == 0 & ref == 1, na.rm = TRUE)

  cm <- matrix(c(TP, FP, FN, TN), nrow = 2, byrow = TRUE,
```

```r
               dimnames = list(Predicted = c("Positive", "Negative"),
                               Actual = c("Positive", "Negative")))

  # Calculate as NUMERIC values first, then format
  sensitivity_num <- TP / (TP + FN)
  specificity_num <- TN / (TN + FP)
  ppv_num <- TP / (TP + FP)
  npv_num <- TN / (TN + FN)

  # Handle division by zero for numeric calculations
  if (is.nan(sensitivity_num) || is.infinite(sensitivity_num)) sensitivity_num <- 0
  if (is.nan(specificity_num) || is.infinite(specificity_num)) specificity_num <- 0
  if (is.nan(ppv_num) || is.infinite(ppv_num)) ppv_num <- 0
  if (is.nan(npv_num) || is.infinite(npv_num)) npv_num <- 0

  # Calculate likelihood ratios using NUMERIC values
  lr_pos <- if (specificity_num == 0) NA else sensitivity_num / (1 - specificity_num)
  lr_neg <- if (specificity_num == 0) NA else (1 - sensitivity_num) / specificity_num

  # Handle infinite/NaN likelihood ratios
  if (is.infinite(lr_pos) || is.nan(lr_pos)) lr_pos <- NA
  if (is.infinite(lr_neg) || is.nan(lr_neg)) lr_neg <- NA

  # Calculate CUI using numeric values
  cui_pos <- sensitivity_num * ppv_num
  cui_neg <- specificity_num * npv_num

  # format for display (but return numeric values for calculations)
  return(list(
    ConfusionMatrix = cm,
    Sensitivity = round(sensitivity_num, 2),
    Specificity = round(specificity_num, 2),
    PPV = round(ppv_num, 2),
    NPV = round(npv_num, 2),
    LR_Pos = if (is.na(lr_pos)) NA else round(lr_pos, 2),
    LR_Neg = if (is.na(lr_neg)) NA else round(lr_neg, 2),
    CUI_Pos = round(cui_pos, 2),
    CUI_Neg = round(cui_neg, 2)
  ))
}
```

```r
# Calculate indices with threshold
calc_indices_with_threshold <- function(predictor, ref, threshold, predictor_name,
                                        threshold_type, disorder_type, predictor_order) {
  pred_binary <- ifelse(predictor > threshold, 1, 0)
  indices <- calc_all_indices(pred_binary, ref)

  # Extract TP, TN, FP, FN from the confusion matrix
  TP <- indices$ConfusionMatrix[1, 1] # True Positive
  FN <- indices$ConfusionMatrix[2, 1] # False Negative
  FP <- indices$ConfusionMatrix[1, 2] # False Positive
  TN <- indices$ConfusionMatrix[2, 2] # True Negative

  # Calculate Diagnostic Odds Ratio (DOR) using (TP * TN) / (FP * FN)
  dor <- if (FP == 0 || FN == 0) NA else round((TP * TN) / (FP * FN), 2)
```

```r
  return(list(
    Predictor = predictor_name,
    Disorder = disorder_type,
    Cut_Off = round(threshold, 1),
    ConfusionMatrix = indices$ConfusionMatrix,
    Sensitivity = round(indices$Sensitivity, 2),
    Specificity = round(indices$Specificity, 2),
    PPV = round(indices$PPV, 2),
    NPV = round(indices$NPV, 2),
    LR_Pos = round(indices$LR_Pos, 2),
    LR_Neg = round(indices$LR_Neg, 2),
    CUI_Pos = round(indices$CUI_Pos, 2),
    CUI_Neg = round(indices$CUI_Neg, 2),
    AUC = round(pROC::auc(pROC::roc(ref, predictor, quiet = TRUE)), 2),
    Diagnostic_OR = dor,
    Predictor_Order = predictor_order
  ))
}
```

```r
# Function to plot ROC curves in a 2x2 grid with Youden's Index
plot_roc_curves <- function(data, predictors, outcome, disorder_name) {
  # Tighter margins and square plots
  par(mfrow = c(2, 2),         # 2x2 grid
      mar = c(3.5, 3.5, 2, 1),# Inner margins: bottom, left, top, right
      oma = c(0, 0, 2, 0),     # Outer margins: less padding overall
      pty = "s",               # Square plotting region
      mgp = c(2, 0.6, 0))      # Axis title & label spacing

  colors <- c("red", "darkgreen", "blue", "purple")
  predictor_names <- c("Red Box", "Green Box", "% Red Box Hours", "Typical Weekly Hours")

  for (i in seq_along(predictors)) {
    roc_obj <- pROC::roc(data[[outcome]], data[[predictors[i]]], quiet = TRUE)
    auc_val <- round(pROC::auc(roc_obj), 3)

    coords_df <- pROC::coords(roc_obj, x = "all", ret = c("specificity", "sensitivity"), transpose = FALSE)
    x_vals <- 1 - coords_df$specificity
    y_vals <- coords_df$sensitivity

    plot(x_vals, y_vals, type = "l", col = colors[i], lwd = 2,
         xlim = c(0, 1), ylim = c(0, 1), asp = 1,
         xlab = "1 - Specificity", ylab = "Sensitivity",
         main = paste0(predictor_names[i], " (AUC = ", auc_val, ")"))

    abline(0, 1, lty = 2, col = "gray")
  }

  par(mfrow = c(1, 1))
}
```

```r
# Helper Function for Contingency Table
create_contingency_table <- function(df, predictor, ref, threshold, disorder_name,
                                     predictor_name) {
  pred_binary <- ifelse(predictor > threshold, 1, 0)
  indices <- calc_all_indices(pred_binary, ref)
```

```r
# Extract TP, TN, FP, FN from the confusion matrix
TP <- indices$ConfusionMatrix[1, 1] # True Positive
FN <- indices$ConfusionMatrix[2, 1] # False Negative
FP <- indices$ConfusionMatrix[1, 2] # False Positive
TN <- indices$ConfusionMatrix[2, 2] # True Negative

# Calculate totals
total_positive <- TP + FN
total_negative <- FP + TN
total_pred_negative <- TN + FN
total_pred_positive <- TP + FP
grand_total <- TP + TN + FP + FN

# Calculate Diagnostic Odds Ratio (DOR) using (TP * TN) / (FP * FN)
dor <- if (FP == 0 || FN == 0) NA else (TP * TN) / (FP * FN)

# Create the contingency table
table_data <- data.frame(
  Row = c(
    paste(disorder_name),  # e.g., "GD"
    paste("Non-", disorder_name, sep = ""),  # e.g., "Non-GD"
    "TOTAL",
    "diagnostic accuracy statistics"
  ),
  Pred_Negative = c(
    TN,  # TN: Predicted negative, actual negative
    FN,  # FN: Predicted negative, actual positive
    total_pred_negative,
    paste("NPV =", sprintf("%.2f", indices$NPV))
  ),
  Pred_Positive = c(
    FP,  # FP: Predicted positive, actual negative
    TP,  # TP: Predicted positive, actual positive
    total_pred_positive,
    paste("PPV =", sprintf("%.2f", indices$PPV))
  ),
  Total = c(
    total_negative,
    total_positive,
    grand_total,
    paste("Diagnostic OR =", sprintf("%.2f", dor))
  ),
  Diagnostic_Accuracy = c(
    "",  # Placeholder for first row
    "",  # Placeholder for second row
    "",  # Placeholder for third row
    paste("Se =", sprintf("%.2f", indices$Sensitivity), "\n",
          "Sp =", sprintf("%.2f", indices$Specificity), "\n",
          "LR+ =", sprintf("%.2f", indices$LR_Pos), "\n",
          "LR- =", sprintf("%.2f", indices$LR_Neg))
  ),
  stringsAsFactors = FALSE
)

# Rename columns to match the desired format
```

```r
  colnames(table_data) <- c(
    paste(disorder_name, "status"),  # e.g., "GD status"
    paste(predictor_name, "\n<", sprintf("%.1f", threshold), "hours"),  # e.g., "< 9.5 hours"
    paste(predictor_name, "\n>", sprintf("%.1f", threshold), "hours"),  # e.g., "> 9.5 hours"
    "TOTAL",
    "diagnostic accuracy"
  )

  return(list(
    table = table_data,
    predictor_name = predictor_name,
    disorder_name = disorder_name
  ))
}
```

```r
# Define predictors with their order
predictors <- list(
  list(name = "Red Box (total hrs)", var = "red_box", order = 1),
  list(name = "Green Box (total hrs)", var = "green_box", order = 2),
  list(name = "Green + red hours", var = "green_plus_red", order = 3),
  list(name = "Proportion red box", var = "red_proportion", order = 4),
  list(name = "Typical weekly hours", var = "typical_weekly_hours", order = 5)
)

# Define disorder types
disorders <- list(
  list(name = "GD", var = "GD_status_binary"),
  list(name = "IGD", var = "IGD_status_binary")
)

# Calculate thresholds and indices
diag_results <- list()

for (d in disorders) {
  for (p in predictors) {
    # Create complete case subset for this specific predictor-outcome pair
    complete_cases <- complete.cases(df[, c(p$var, d$var)])

    if (sum(complete_cases) < 10) {
      warning(paste("Insufficient complete cases for", d$name, "vs", p$name))
      next
    }

    predictor_data <- df[[p$var]][complete_cases]
    outcome_data <- df[[d$var]][complete_cases]

    if (sum(outcome_data, na.rm = TRUE) >= 2) {
      roc_obj <- pROC::roc(outcome_data, predictor_data, quiet = TRUE)
      auc_value <- round(pROC::auc(roc_obj), 3)
      roc_threshold <- pROC::coords(roc_obj, "best", ret = "threshold",
                                   best.method = "youden")$threshold

      result_key <- paste(d$name, p$name, "ROC", sep = "_")
      diag_results[[result_key]] <- calc_indices_with_threshold(
        predictor_data, outcome_data, roc_threshold, p$name,
```

```
        "ROC-Optimized (Youden)", d$name, p$order
      )

      diag_results[[result_key]]$AUC <- auc_value

      message(paste("Completed ROC analysis for", p$name, "predicting", d$name,
                    "- AUC:", auc_value))
    } else {
      warning(paste("Not enough positive cases for", d$name,
                    "to perform ROC analysis with", p$name))
    }
  }
}

# Create dataset for plotting (use most complete subset)
plot_vars <- c("red_box", "green_box", "red_proportion", "typical_weekly_hours",
               "GD_status_binary", "IGD_status_binary")
df_complete <- df[complete.cases(df[, plot_vars]), ]
```

```
# Generate Figure 1: ROC Curves for GD (ICD-11)
roc_predictors <- c("red_box", "green_box", "red_proportion", "typical_weekly_hours")
plot_roc_curves(df_complete, roc_predictors, "GD_status_binary", "ICD-11 gaming disorder")
```

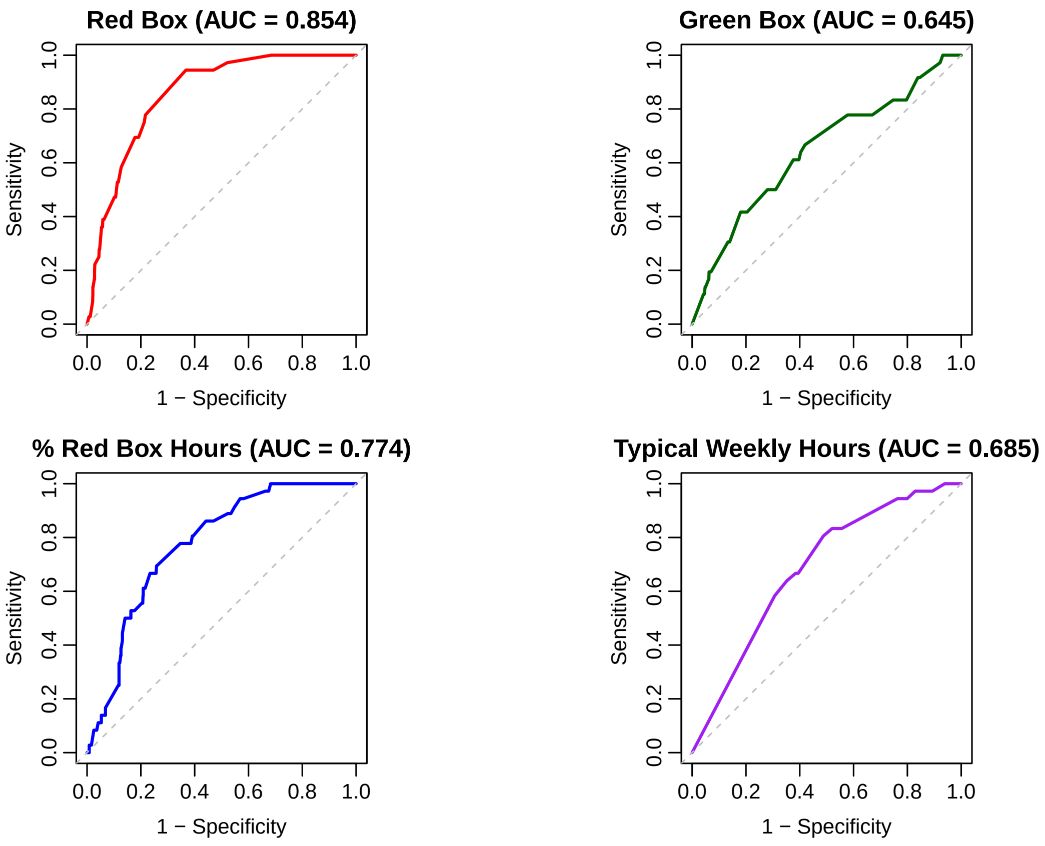

Table 3. Diagnostic Accuracy Indices

```r
# Create diagnostic accuracy table (Table 3)
table_3 <- do.call(rbind, lapply(diag_results, function(x) {
  data.frame(
    Disorder = x$Disorder,
    Predictor = x$Predictor,
    Cut_Off = x$Cut_Off,
    Sensitivity = x$Sensitivity,
    Specificity = x$Specificity,
    PPV = x$PPV,
    NPV = x$NPV,
    LR_Pos = x$LR_Pos,
    LR_Neg = x$LR_Neg,
    CUI_Pos = x$CUI_Pos,
    CUI_Neg = x$CUI_Neg,
    AUC = x$AUC,
    Diagnostic_OR = x$Diagnostic_OR,
    Predictor_Order = x$Predictor_Order,
    stringsAsFactors = FALSE
  )
}))

# Order table by disorder and predictor
table_3 <- table_3[order(table_3$Disorder, table_3$Predictor_Order), ]

# Remove Predictor_Order column
table_3 <- subset(table_3, select = -c(Predictor_Order))

# Create header rows for combining later
header_gd <- data.frame(
  Disorder = "GD",
  Predictor = "ICD-11 gaming disorder",
  Cut_Off = "", Sensitivity = "", Specificity = "", PPV = "", NPV = "",
  LR_Pos = "", LR_Neg = "", CUI_Pos = "", CUI_Neg = "", AUC = "", Diagnostic_OR = "",
  stringsAsFactors = FALSE
)

header_igd <- data.frame(
  Disorder = "IGD",
  Predictor = "DSM-5 internet gaming disorder",
  Cut_Off = "", Sensitivity = "", Specificity = "", PPV = "", NPV = "",
  LR_Pos = "", LR_Neg = "", CUI_Pos = "", CUI_Neg = "", AUC = "", Diagnostic_OR = "",
  stringsAsFactors = FALSE
)

# Split and recombine
gd_rows <- table_3[table_3$Disorder == "GD", ]
igd_rows <- table_3[table_3$Disorder == "IGD", ]

table_3_final <- rbind(gd_rows, igd_rows)

# Save results (if desired, with headers still in)
write.csv(table_3_final,
          file.path(project_dirs$dir_paths$manuscript_files,
```

```
                    "Table 3 - Diagnostic Accuracy Indices.csv"),
          row.names = FALSE)
```

```
table_3_display <- table_3_final[, -1]

# Calculate group row ranges
n_gd <- nrow(gd_rows)
n_igd <- nrow(igd_rows)

kbl(table_3_display,
    caption = "Table 3. Diagnostic accuracy indices for gaming time measures",
    col.names = c("Predictor", "Cut-off", "Se", "Sp", "PPV", "NPV", "LR+", "LR-",
                  "CUI+", "CUI-", "AUC", "DOR"),
    booktabs = TRUE,
    longtable = TRUE,
    row.names = FALSE,
    escape = FALSE,
    align = c("l", rep("c", 11))) %>%
  kable_styling(latex_options = c("striped", "hold_position", "repeat_header"),
                font_size = 9) %>%  # Use full width
  column_spec(1, width = "4cm") %>%  # Only specify the first column width
  group_rows("ICD-11 Gaming Disorder", start_row = 1, end_row = n_gd) %>%
  group_rows("DSM-5 Internet Gaming Disorder", start_row = n_gd + 1, end_row = n_gd + n_igd) %>%
  footnote(
    general = "Se = Sensitivity; Sp = Specificity; PPV = Positive Predictive Value;
    NPV = Negative Predictive Value; LR+ = Positive Likelihood Ratio;
    LR- = Negative Likelihood Ratio; CUI+ = Positive Clinical Utility Index;
    CUI- = Negative Clinical Utility Index; AUC = Area Under the Curve;
    DOR = Diagnostic Odds Ratio.",
    threeparttable = TRUE,
    escape = FALSE
  )
```

Table 3. Diagnostic accuracy indices for gaming time measures

Predictor	Cut-off	Se	Sp	PPV	NPV	LR+	LR-	CUI+	CUI-	AUC	DOR
**ICD-11 Gaming Disorder**											
Red Box (total hrs)	9.5	0.94	0.63	0.08	1.00	2.57	0.09	0.07	0.63	0.855	29.33
Green Box (total hrs)	20.5	0.67	0.58	0.05	0.98	1.59	0.57	0.03	0.57	0.646	2.77
Green + red hours	35.5	0.75	0.70	0.08	0.99	2.53	0.36	0.06	0.70	0.776	7.11
Proportion red box	34.1	0.69	0.74	0.08	0.99	2.69	0.41	0.06	0.73	0.774	6.53
Typical weekly hours	24.5	0.81	0.51	0.05	0.99	1.65	0.38	0.04	0.51	0.685	4.35
**DSM-5 Internet Gaming Disorder**											
Red Box (total hrs)	5.5	0.88	0.52	0.21	0.97	1.82	0.23	0.19	0.50	0.757	7.85
Green Box (total hrs)	28.5	0.48	0.74	0.22	0.90	1.85	0.70	0.10	0.67	0.616	2.63
Green + red hours	38.5	0.58	0.76	0.27	0.92	2.41	0.55	0.15	0.70	0.701	4.36
Proportion red box	30.9	0.64	0.67	0.22	0.92	1.92	0.54	0.14	0.62	0.688	3.56
Typical weekly hours	24.5	0.71	0.53	0.19	0.93	1.53	0.54	0.13	0.49	0.654	2.86

Note:
Se = Sensitivity; Sp = Specificity; PPV = Positive Predictive Value; NPV = Negative Predictive Value; LR+ = Positive Likelihood Ratio; LR- = Negative Likelihood Ratio; CUI+ = Positive Clinical Utility Index; CUI- = Negative Clinical Utility Index; AUC = Area Under the Curve; DOR = Diagnostic Odds Ratio.

SUPPLEMENTARY RESULTS

Table S1. Sample Characteristics - By DSM-5 symptom criteria

```
supp_table_1 <- create_descriptive_table(df, group_var = "IGD_status", group_value = "IGD")

write.csv(supp_table_1,
          file.path(project_dirs$dir_paths$supplementary_files,
                    "Supplementary Table 1 - Sample Characteristics by IGD Status.csv"),
          row.names = FALSE)

kbl(supp_table_1,
    caption = "Supplementary Table 1. Sample characteristics, stratified by DSM-5
    internet gaming disorder status",
    col.names = c("Characteristics", "Total (N=1,149)", "Non-IGD (N=999)", "IGD (N=150)", "t/X2", "p"),
    booktabs = TRUE,
    longtable = TRUE,
    row.names = FALSE,
    escape = TRUE,
    align = c("l", "r", "r", "r", "r", "r")) %>%
  kable_styling(latex_options = c("striped", "hold_position", "repeat_header"),
                font_size = 9) %>%
  footnote(general = "Internet gaming disorder (IGD) was classified by endorsement
           of 5 or more items from IGDT-10. BIS-15 = 15-item Barratt Impulsivity Scale.
           DASS-21 = 21-item Depression, Anxiety and Stress Scale.",
           threeparttable = TRUE,
           escape = TRUE)
```

Supplementary Table 1. Sample characteristics, stratified by DSM-5 internet gaming disorder status

Characteristics	Total (N=1,149)	Non-IGD (N=999)	IGD (N=150)	t/X2	p
Age, years; mean (SD)	25 (3.5)	25 (3.5)	24.9 (3.2)	-0.5	0.642
Nationality	–	–	–	N/A	0.785
American	615 (53.5%)	535 (53.6%)	80 (53.3%)	-	-
Asian	61 (5.3%)	51 (5.1%)	10 (6.7%)	-	-
Australian or New Zealand	46 (4.0%)	42 (4.2%)	4 (2.7%)	-	-
European	335 (29.2%)	289 (28.9%)	46 (30.7%)	-	-
Other	92 (8.0%)	82 (8.2%)	10 (6.7%)	-	-
Employment status	–	–	–	N/A	0.066
Casual	49 (4.3%)	42 (4.2%)	7 (4.7%)	-	-
Full-time	451 (39.3%)	393 (39.3%)	58 (38.7%)	-	-
Not Employed	266 (23.2%)	220 (22.0%)	46 (30.7%)	-	-
Part-time	176 (15.3%)	160 (16.0%)	16 (10.7%)	-	-
Retired	2 (0.2%)	1 (0.1%)	1 (0.7%)	-	-
Studying	205 (17.8%)	183 (18.3%)	22 (14.7%)	-	-
Highest educational level attained	–	–	–	N/A	0.463
#NULL!	1 (0.1%)	1 (0.1%)	0 (0.0%)	-	-
Further (e.g. Apprentice, TAFE)	77 (6.7%)	64 (6.4%)	13 (8.7%)	-	-
Higher (ie. Undergraduate, College)	583 (50.7%)	503 (50.4%)	80 (53.3%)	-	-
Other (please Specify)	21 (1.8%)	20 (2.0%)	1 (0.7%)	-	-
Postgraduate (e.g. Masters, Ph.D)	78 (6.8%)	72 (7.2%)	6 (4.0%)	-	-
Secondary/High School	389 (33.9%)	339 (33.9%)	50 (33.3%)	-	-
Gaming time measures	–	–	–	-	-

Supplementary Table 1. Sample characteristics, stratified by DSM *(continued)*

Characteristics	Total (N=1,149)	Non-IGD (N=999)	IGD (N=150)	t/X2	p
Red box hours; mean (SD)	9.1 (8.8)	8 (7.7)	16.2 (11.8)	8.3	<.001
Green box hours; mean (SD)	22.6 (11.6)	21.9 (11.1)	27.7 (13.8)	4.9	<.001
Total green & red hours; mean (SD)	31.7 (17.1)	29.9 (15.5)	43.9 (21.9)	7.6	<.001
Proportion red box hours (%), mean (SD)	27.3 (16.7)	26 (16.5)	35.9 (15.1)	7.4	<.001
Typical weekly hours; mean (SD)	23.7 (6.5)	23.2 (6.5)	26.6 (5.7)	6.8	<.001
Impulsivity (BIS-15)	–	–	–	-	-
Non-planning; mean (SD)	11.9 (3.5)	11.7 (3.5)	12.9 (3.8)	3.6	<.001
Motor; mean (SD)	10.2 (3.2)	9.9 (3.0)	12.1 (3.6)	7.2	<.001
Attentional; mean (SD)	10.7 (3.0)	10.4 (2.9)	12.3 (3.3)	6.6	<.001
Total score; mean (SD)	32.7 (7.5)	32.1 (7.2)	37.3 (8.2)	7.4	<.001
Psychological distress (DASS-21)	–	–	–	-	-
Stress; mean (SD)	7.4 (5.2)	6.8 (5.0)	11.4 (4.9)	10.6	<.001
Anxiety; mean (SD)	5.3 (4.7)	4.8 (4.4)	8.9 (5.1)	9.5	<.001
Depression; mean (SD)	7.7 (6.1)	7.1 (5.9)	11.7 (6.0)	8.8	<.001
Total score; mean (SD)	20.4 (14.6)	18.6 (13.9)	32 (14.3)	10.7	<.001

Note:
makecell[l]Internet gaming disorder (IGD) was classified by endorsement of 5 or more items from IGDT-10. BIS-15 = 15-item Barratt Impulsivity Scale. DASS-21 = 21-item Depression, Anxiety and Stress Scale.

Table S2. Zero Order Correlation Matrix.

```
# Define required variables for correlation analysis
required_vars_for_analysis <- c(
  "typical_weekly_hours", "red_box", "green_box", "IGD_status_binary",
  "GD_status_binary", "total_symptoms", "non_planning", "attentional",
  "motor", "total_impulsivity", "stress", "anxiety", "depression",
  "DASS_total", "age", "nationality_recoded", "employment_recoded",
  "education_recoded"
)

# Only use variables that actually exist in the dataset
existing_analysis_vars <- required_vars_for_analysis[required_vars_for_analysis
                                                     %in% colnames(df)]

df_complete <- df[complete.cases(df[, existing_analysis_vars]), ]
zero_cor_mat <- zero_order_cor_matrix(df_complete)

write.csv(zero_cor_mat,
          file.path(project_dirs$dir_paths$supplementary_files,
                    "Supplementary Table 2 - Zero-order correlation matrix.csv"),
          row.names = TRUE)

upper_triangle_matrix_2 <- zero_cor_mat
upper_triangle_matrix_2[lower.tri(upper_triangle_matrix_2, diag = FALSE)] <- ""

corr_df_2 <- as.data.frame(upper_triangle_matrix_2)

new_names<- c(
  "Weekly hours", "Red Box hrs", "Green Box hrs", "IGDT items", "GD status", "IGD status",
  "BIS Non-Planning", "BIS Attentional", "BIS Motor", "DASS Stress", "DASS Anxiety", "DASS Depression",
```

```r
  "Age", "Nat.", "Emp.", "Ed."
)

if(nrow(corr_df_2) <= length(new_names)) {
  rownames(corr_df_2) <- new_names[1:nrow(corr_df_2)]
}

kbl(corr_df_2,
                caption = "Supplementary Table 2. Zero-order correlation matrix",
                booktabs = TRUE,
                longtable = TRUE,
                col.names = if(ncol(corr_df_2) <= length(new_names)) {
                  new_names[1:ncol(corr_df_2)]
                } else {
                  c(new_names, colnames(corr_df_2)[(
                    length(new_names)+1):ncol(corr_df_2)])
                }) %>%
  kable_styling(latex_options = c("striped", "hover", "condensed", "responsive"),
                font_size = 8) %>%
  footnote(general = "* p < 0.05; ** p < 0.001; Nat. = Nationality; Emp. = Employment; Ed. = Education.",
           threeparttable = TRUE,
           escape = FALSE) %>%
  column_spec(1, width = "2.8cm") %>%
  column_spec(2:16, width = "0.85cm") %>%
  landscape()
```

Supplementary Table 2. Zero-order correlation matrix

	Weekly hours	Red Box hrs	Green Box hrs	IGDT items	GD status	IGD status	BIS Non-Planning	BIS Attentional	BIS Motor	DASS Stress	DASS Anxiety	DASS Depression	Age	Nat.	Emp.	Ed.
Weekly hours	1.00	0.35**	0.65**	0.26**	0.11**	0.18**	0.16**	0.04	0.06	0.12**	0.14**	0.15**	-0.09*	-0.04	0.08*	0.12**
Red Box hrs		1.00	0.40**	0.41**	0.25**	0.32**	0.15**	0.19**	0.18**	0.29**	0.31**	0.29**	-0.11**	-0.09*	0.11**	0.05
Green Box hrs			1.00	0.21**	0.10**	0.17**	0.15**	0.04	0.05	0.09*	0.11**	0.15**	-0.06*	-0.09*	0.06*	0.11**
IGDT items				1.00	0.46**	0.79**	0.14**	0.32**	0.33**	0.45**	0.43**	0.39**	-0.05	-0.02	-0.05	0.02
GD status					1.00	0.43**	0.06	0.20**	0.21**	0.20**	0.17**	0.17**	-0.01	0.02	0.02	-0.04
IGD status						1.00	0.11**	0.21**	0.24**	0.29**	0.30**	0.25**	-0.01	-0.01	-0.03	-0.03
BIS Non-Planning							1.00	0.32**	0.37**	0.19**	0.15**	0.25**	-0.06*	-0.01	0.10**	0.12**
BIS Attentional								1.00	0.51**	0.49**	0.41**	0.41**	-0.12**	0.06*	0.01	0.00
BIS Motor									1.00	0.42**	0.36**	0.31**	-0.09*	0.07*	-0.02	-0.02
DASS Stress										1.00	0.80**	0.77**	-0.05	0.01	-0.03	0.02
DASS Anxiety											1.00	0.68**	-0.07*	-0.02	-0.04	0.04
DASS Depression												1.00	0.00	-0.00	-0.03	0.07*
Age													1.00	-0.06*	-0.42**	-0.13**
Nat.														1.00	-0.02	-0.15**
Emp.															1.00	0.04
Ed.																1.00

Note:
* $p < 0.05$; ** $p < 0.001$; Nat. = Nationality; Emp. = Employment; Ed. = Education.

Figure S1. ROC Curve Analysis (IGD).

```r
# Generate Supplementary Figure: ROC Curves for IGD (DSM-5)
plot_roc_curves(df_complete, roc_predictors, "IGD_status_binary",
                "DSM-5 internet gaming disorder")
```

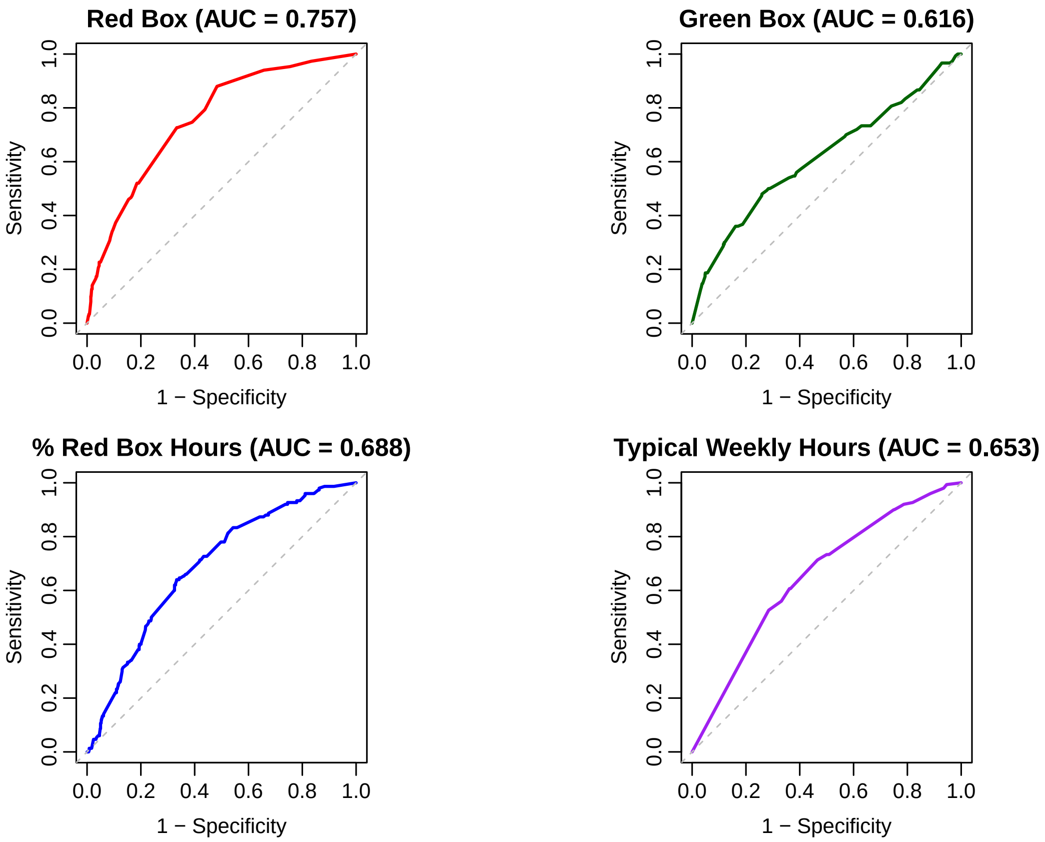

Contingency Tables (not displayed)

```r
# Generate contingency tables (supplementary)
contingency_tables <- list()

for (d in disorders) {
  for (p in predictors) {
    if (sum(d$data, na.rm = TRUE) >= 2) {
      roc_obj <- pROC::roc(d$data, p$data, quiet = TRUE)
      roc_threshold <- pROC::coords(roc_obj, "best", ret = "threshold",
                                    best.method = "youden")$threshold

      contingency_table <- create_contingency_table(
        df, p$data, d$data, roc_threshold, d$name, p$name
      )
```

```r
      result_key <- paste(d$name, p$name, "Contingency", sep = "_")
      contingency_tables[[result_key]] <- contingency_table
    }
  }
}

# Save contingency tables
for (ct in contingency_tables) {
  file_name <- sprintf("Supplementary_Table_Contingency_%s_%s.csv",
                       ct$disorder_name, ct$predictor_name)
  write.csv(ct$table, file_name, row.names = FALSE)
}
```

REFERENCES

1. Grant JE, Potenza MN, Weinstein A, Gorelick DA. Introduction to behavioral addictions. The American Journal of Drug and Alcohol Abuse. 2010;36(5):233-41. https://doi.org/10.3109/00952990.2010.491884.

2. King DL, Billieux J, Carragher N, Delfabbro PH. Face validity evaluation of screening tools for gaming disorder: scope, language, and overpathologizing issues. Journal of Behavioral Addictions. 2020;9(1):1-13. https://doi.org/10.1556/2006.2020.00001.

3. King DL, Nogueira-López A, Galanis CR, Hamamura T, Bäcklund C, Giardina A, et al. Reconsidering item response categories in gaming disorder symptoms. Journal of Behavioral Addictions. 2023;12(4):873-7. https://doi.org/10.1556/2006.2023.00070.

4. King DL, Chamberlain SR, Carragher N, Billieux J, Stein D, Mueller K, et al. Screening and assessment tools for gaming disorder: a comprehensive systematic review. Clinical Psychology Review. 2020;77:101831. https://doi.org/10.1016/j.cpr.2020.101831.

5. Yoon S, Yang Y, Ro E, Ahn WY, Kim J, Shin SH, Chey J, Choi KH. Reliability, and convergent and discriminant validity of gaming disorder scales: A meta-analysis. Frontiers in Psychology. 2021;12:764209. https://doi.org/10.3389/fpsyg.2021.764209.

6. King DL, Billieux J, Delfabbro PH. Red Box, Green Box: a self-report behavioral frequency measurement approach for behavioral addictions research. Journal of Behavioral Addictions. 2024;13(1):21-4. https://doi.org/10.1556/2006.2023.00079.

7. Stevens MWR, Radünz M, Galanis C, Quinney B, Zajac I, Billieux J, Delfabbro PH, King, DL. Red box, green box: A self-report behavioral frequency measurement approach for behavioral addictions research. [dataset]. Mendeley. 2025.

8. Király O, Sleczka P, Pontes HM, Urbán R, Griffiths MD, Demetrovics Z. Validation of the ten-item internet gaming disorder test (IGDT-10) and evaluation of the nine DSM-5 internet gaming disorder criteria. Addictive behaviors. 2017;64:253-60.

9. American Psychiatric Association. Diagnostic and Statistical Manual of Mental Disorders (fifth ed. text revision). 2022. https://psychiatryonline.org/doi/full/10.5555/appi.books.9780890425787.

10. World Health Organization. ICD-11 for Mortality and Morbidity Statistics 2025-01. 6C51 Gaming Disorder. WHO, Geneva. https://icd.who.int/browse/2025-01/mms/en#1448597234.

11. Robin X, Turck N, Hainard A, Tiberti N, Lisacek F, Sanchez JC, Müller M. pROC: an open-source package for R and S+ to analyze and compare ROC curves. BMC bioinformatics. 2011;12:1-8. https://doi.org/10.1186/1471-2105-12-77.

12. Stevens MWR, Dorstyn D, Delfabbro PH, King DL. Global prevalence of gaming disorder: A systematic review and meta-analysis. Australian & New Zealand Journal of Psychiatry. 2021;55(6):553-68. https://doi.org/10.1177/0004867420962851.

13. Stevens MWR, Dorstyn D, Delfabbro PH, King DL. Corrigendum to: Stevens, MWR, Dorstyn, D., Delfabbro, PH, & King, DL (2021). Global prevalence of gaming disorder: A systematic review and meta-analysis. Australian and New Zealand Journal of Psychiatry, 55, 553–568. Australian & New Zealand Journal of Psychiatry. 2023;57(6):92. https://doi.org/10.1177/00048674221137011.
